# The microbial dimension of submarine groundwater discharge: current challenges and future directions

**DOI:** 10.1093/femsre/fuab010

**Published:** 2021-02-04

**Authors:** Clara Ruiz-González, Valentí Rodellas, Jordi Garcia-Orellana

**Affiliations:** Institut de Ciències del Mar (ICM-CSIC), Passeig Marítim de la Barceloneta, 37-49, E08003 Barcelona, Spain; Institut de Ciència i Tecnologia Ambientals (ICTA-UAB), Universitat Autònoma de Barcelona, E08193 Bellaterra, Spain; Institut de Ciència i Tecnologia Ambientals (ICTA-UAB), Universitat Autònoma de Barcelona, E08193 Bellaterra, Spain; Departament de Física, Universitat Autònoma de Barcelona, E08193 Bellaterra, Spain

**Keywords:** aquatic prokaryotic communities, submarine groundwater discharge, coastal aquifers, subterranean estuaries, microbial diversity and ecology, ultrasmall prokaryotes

## Abstract

Despite the relevance of submarine groundwater discharge (SGD) for ocean biogeochemistry, the microbial dimension of SGD remains poorly understood. SGD can influence marine microbial communities through supplying chemical compounds and microorganisms, and in turn, microbes at the land–ocean transition zone determine the chemistry of the groundwater reaching the ocean. However, compared with inland groundwater, little is known about microbial communities in coastal aquifers. Here, we review the state of the art of the microbial dimension of SGD, with emphasis on prokaryotes, and identify current challenges and future directions. Main challenges include improving the diversity description of groundwater microbiota, characterized by ultrasmall, inactive and novel taxa, and by high ratios of sediment-attached versus free-living cells. Studies should explore microbial dynamics and their role in chemical cycles in coastal aquifers, the bidirectional dispersal of groundwater and seawater microorganisms, and marine bacterioplankton responses to SGD. This will require not only combining sequencing methods, visualization and linking taxonomy to activity but also considering the entire groundwater–marine continuum. Interactions between traditionally independent disciplines (e.g. hydrogeology, microbial ecology) are needed to frame the study of terrestrial and aquatic microorganisms beyond the limits of their presumed habitats, and to foster our understanding of SGD processes and their influence in coastal biogeochemical cycles.

## Teaser

The authors review the available literature on the microbial aspects of submarine groundwater discharge, from the freshwater aquifers to the coastal ocean, and identify current challenges and future directions to foster knowledge on microbial ecology at the land–ocean interface.

## INTRODUCTION

Aquifers contain a significant portion of the Earth's freshwater (∼23 × 10^6^ km^3^; Gleeson *et al*. 2016) and a large fraction of the global microbial diversity (Magnabosco *et al*. 2018), yet they have been much less studied than surface aquatic ecosystems. Like in deep soils, deep marine sediments or the dark bathypelagic ocean (Acinas *et al*. 2019; Brewer *et al*. 2019; Wörmer *et al*. 2019), the absence of photosynthesis-derived labile organic carbon has forced groundwater organisms (mostly prokaryotic microorganisms—Bacteria and Archaea) to develop diverse strategies to live or persist (Griebler and Lueders 2009). For example, heterotrophic groundwater microorganisms can use ancient organic carbon from rocks (Nowak *et al*. 2017), allochthonous organic carbon such as plant-derived material (Taubert *et al*. 2019), or can degrade organic contaminants (Griebler and Lueders 2009; Smith *et al*. 2015). Other groundwater microbes are capable of diverse chemoautotrophic metabolisms that allow them to fix inorganic carbon using energy from the oxidation of substrates such as ammonium, nitrite and reduced iron and sulfur compounds (Herrmann *et al*. 2015; Anantharaman *et al*. 2016; Kumar *et al*. 2017, 2018; Probst *et al*. 2017, 2018). Groundwater microorganisms, however, are largely under-represented in global diversity catalogs, as for example less than 2% of the public 16S ribosomal RNA (rRNA) gene sequences (i.e. the most common taxonomic marker for identifying prokaryotic taxa, Appendix 1) were calculated to derive from groundwater organisms (Schloss *et al*. 2016). The magnitude of this knowledge gap has become evident in recent years: 47 previously unknown phyla were found in a single aquifer (Anantharaman *et al*. 2016), highlighting a tremendous potential for taxonomic and metabolic discovery in subsurface ecosystems.

**Figure 1.**
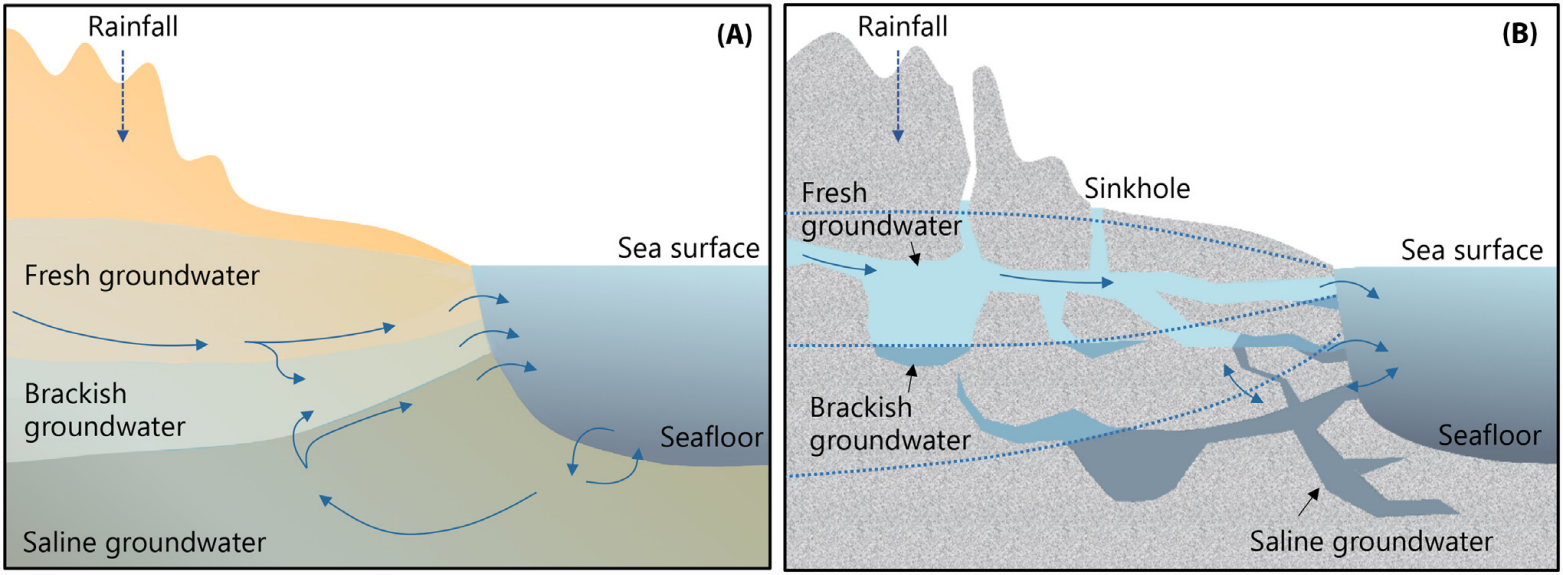
Simplified scheme of SGD, including diffusive seepage through unconsolidated sediments **(A)** and point-sourced discharge, as in karstic or volcanic aquifers **(B)**. Most of the groundwater flow in the latter systems is likely to occur through conduits, but it can also occur through the porous and thinly fissured rocks, developing mixing zones similar to systems with unconsolidated sediments (dotted lines) (B). (All illustrations by C. Ruiz-González).

Among aquifers, those located in coastal areas are essential freshwater sources for humanity given that half of the global population is concentrated along the oceans' shoreline (Small and Nichols 2003; Barragán and de Andrés 2015; Michael *et al*. 2017). Coastal aquifers are also vulnerable ecosystems because the intense anthropogenic pressure is causing pollution and salinization of coastal groundwater resources worldwide (Ferguson and Gleeson 2012; Werner *et al*. 2013). Their direct connection to the ocean through permeable sediments or rocks (Fig. 1) allows the discharge of groundwater, a process known as submarine groundwater discharge (SGD; Box 1), but also the entrance of seawater. This often results in an area of active mixing within the aquifer, referred to as 'subterranean estuary' (Moore 1999; Duque, Michael and Wilson 2020; Box 1). Globally, SGD fluxes (∼10^14^ m^3^ yr^−1^) have been estimated to be 3–4 times greater than the riverine water fluxes into the oceans (Kwon *et al*. 2014), representing a very important source of dissolved nutrients and other chemical compounds (e.g. metals, carbon, pollutants, greenhouse gases) to the coastal ocean (Moore 2010; Rodellas *et al*. 2015; Cho *et al*. 2018; Mayfield *et al*. 2021). SGD can comprise meteoric groundwater originating through aquifer recharge (fresh groundwater), seawater infiltrated into coastal aquifers and sediments (saline groundwater), or a mixture of both (brackish groundwater) (Moore 2010; Fig. 1). At the global scale, SGD is mainly composed of saline groundwater, with fresh groundwater representing only <1% of total SGD but sometimes being the dominant component at a more local scale (Luijendijk, Gleeson and Moosdorf 2020). In general, most SGD to the coastal ocean occurs through slow and diffusive seeps through permeable sediments (Fig. 1A), but in systems with fractured rocks or preferential flow paths (e.g. karstic carbonate or volcanic systems) groundwater can also be transferred to the coastal ocean through point-sourced seeps or submarine springs (Tovar-Sánchez *et al*. 2014; Fig. 1B).

BOX 1. **Terms and definitions**

**Coastal aquifers:** Coastal aquifers are frequently defined as permeable geological units in the subsurface that can transmit significant flows of water and that are connected with the sea (Duque *et al*. 2020). Coastal aquifers consist of unconsolidated surficial deposits (created by alluvial, lacustrine, deltaic, glacial or eolian processes; e.g. Fig. 1A), permeable rocks (e.g. basalts, sandstones or limestones) or heavily fractured systems (e.g. karstified carbonate rocks or volcanic systems; Fig. 1B), where cavities and pores between mineral grains provide open spaces for water storage and transmission (Freeze and Cherry 1979).

**Subterranean estuary:** The term subterranean estuary was initially introduced to emphasize the importance of biogeochemical reactions occurring within coastal aquifers, where meteoric groundwater mixes with the seawater that has entered into the coastal aquifer (Moore, 1999). The subterranean estuary is now considered as the part of the coastal aquifer that actively interacts with the ocean, including thus the area of fresh groundwater that is connected to the sea (i.e. groundwater that could discharge to the ocean), the mixing zone between meteoric and marine groundwater, and the permeable sediments filled with completely saline groundwater that may exchange with overlying marine waters (Duque *et al*. 2020).

**Submarine groundwater discharge (SGD):** Groundwater is often considered as 'any water in the ground', regardless of its composition or origin, and thus it is synonymous with porewater (Burnett *et al*. 2003). Groundwater can thus consist of water of any salinity, ranging from freshwater from recharged aquifers on land to seawater that has invaded the subterranean estuary. Based on this concept, SGD is commonly defined as 'the flow of water through continental and insular margins from the seabed to the coastal ocean, regardless of fluid composition or driving force' (Burnett *et al*. 2003; Moore 2010; Taniguchi *et al*. 2019). SGD incorporates thus water flows with different characteristics and is driven by different forces, including discharge of fresh groundwater driven by the terrestrial hydraulic gradient, exchange of seawater driven by the movement of the freshwater–saltwater interface, or circulation of seawater driven by tidal inundation or wave run-up, among others (Santos *et al*. 2012; Robinson *et al*. 2018).

Coastal aquifers and SGD are of special interest from a microbiological perspective. First, coastal aquifers are located at the land–ocean interface, a transition zone with steep physicochemical gradients (e.g. freshwater–seawater, oxic–anoxic) that promote the establishment of spatially heterogeneous microbial communities (Héry *et al*. 2014; Beck *et al*. 2017; Seibert *et al*. 2020). Second, given that groundwater microbes control the cycling of nutrients, carbon and trace elements in aquifers (Hunter, Wang and Van Cappellen 1998; Griebler and Lueders 2009; Flynn *et al*. 2013; Griebler and Avramov 2015; Meckenstock *et al*. 2015; Hoffman *et al*. 2020), microbial activity in subterranean estuaries will determine the chemical composition of the groundwater reaching the ocean, but little is known regarding the metabolic functions of microorganisms in coastal aquifers (Santoro 2010; Seibert *et al*. 2020). Finally, despite the relevance of bacterioplankton communities for marine food webs and biogeochemical processes (e.g. Whitman, Coleman and Wiebe 1998; Gasol *et al*. 2008), only a few studies have addressed marine bacterial taxonomic and functional responses to SGD (see references in Lecher and Mackey 2018). Improving our understanding on the microbial players along the entire aquatic continuum from freshwater aquifers to the coastal ocean is essential to gain insight into the complex processes underlying coastal groundwater biogeochemistry, the potential variations in these processes due to global change, and their influence on marine ecosystems.

The aim of this review is to provide an overview of the current knowledge on microbial ecology along the land–ocean transition zone connected by SGD. We first identify current challenges for the study of coastal groundwater microbiota based on recent discoveries in inland aquifers, which have been much more intensely studied than coastal groundwater systems (see the section ‘Challenges for assessing microbial diversity and their function in the coastal subsurface: lessons learned from inland aquifer research'). Second, we review the available literature on microbial diversity and ecology along subterranean estuaries and the adjacent coast influenced by SGD (see the section ‘Microbial ecology at the terrestrial–marine interface connected by groundwater'). Finally, we provide future research directions and strategies that may foster our understanding of the microbial dimension underlying SGD processes (see the section ‘Future directions and research avenues').

## CHALLENGES FOR ASSESSING MICROBIAL DIVERSITY AND THEIR FUNCTION IN THE COASTAL SUBSURFACE: LESSONS LEARNED FROM INLAND AQUIFER RESEARCH

**Figure 2.**
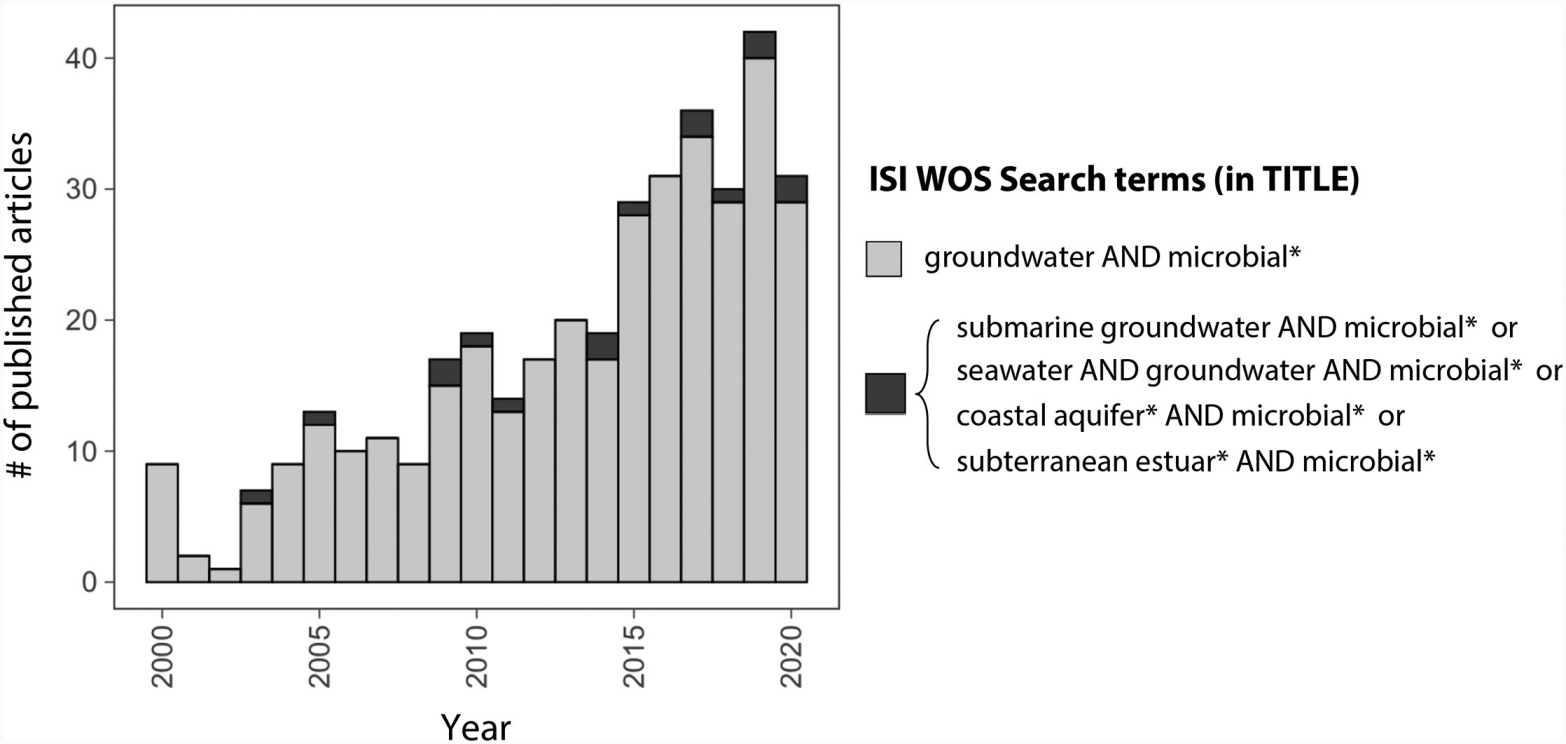
Rising interest in groundwater microbiology over the last two decades, and scarcity of studies targeting coastal aquifers. Number of articles published per year (2000–2020) using the search terms ‘groundwater AND microbial*' (gray, *n* = 369) or adding ‘submarine', ‘seawater', ‘coastal aquifer*' or ‘subterranean estuar*' to the search term set (black, *n* = 16) in the title, as indexed in the ISI Web of Science. Only articles in English were considered (search performed in August 2020).

Despite the growing interest in inland groundwater microbiology in the last decades, coastal aquifers have received comparably little attention. A simple search in the ISI Web of Science database looking for articles published between 2000 and 2020 containing ‘groundwater AND microbial*' in the title retrieves 369 studies that show a clear increasing trend over time (Fig. 2). Conversely, only 16 articles are recovered if we include terms such as ‘submarine', ‘seawater', ‘subterranean estuar*' or ‘coastal aquifer', highlighting a wide gap of knowledge on the marine–groundwater interface from a microbial perspective. This growing body of inland groundwater research provides, nonetheless, a solid basis for anticipating the main challenges that the study of the microbiology in subterranean estuaries might face. Based on this knowledge, in this section we identify four main challenges related to groundwater microbial idiosyncrasies that future investigations should take into account for an accurate understanding of microbial ecology at the land–ocean interface.

Additional challenges related to sampling the subsurface or SGD sources are not discussed in this review. For example, aquifers need to be accessed through wells, piezometers, boreholes, cores or excavations. This not only makes the collection of groundwater and aquifer solids technically and economically challenging (Lehman 2007), but also precludes an accurate characterization of the geologic, physicochemical and biological 3D heterogeneity of subsurface ecosystems (Robinson *et al*. 2018; Smith *et al*. 2018, Folch *et al*. 2020). Similarly, SGD is a much more diffuse and heterogeneous source of water to the ocean than riverine discharge, which translates into many uncertainties associated with the magnitude and spatiotemporal variability of this process (Burnett *et al*. 2006). Current and future efforts to improve the characterization of aquifers and groundwater seeps to the ocean will be instrumental for developing the research lines identified in this review.

### Novel groundwater microbial taxa can be missed by current sequencing technologies

**Figure 3.**
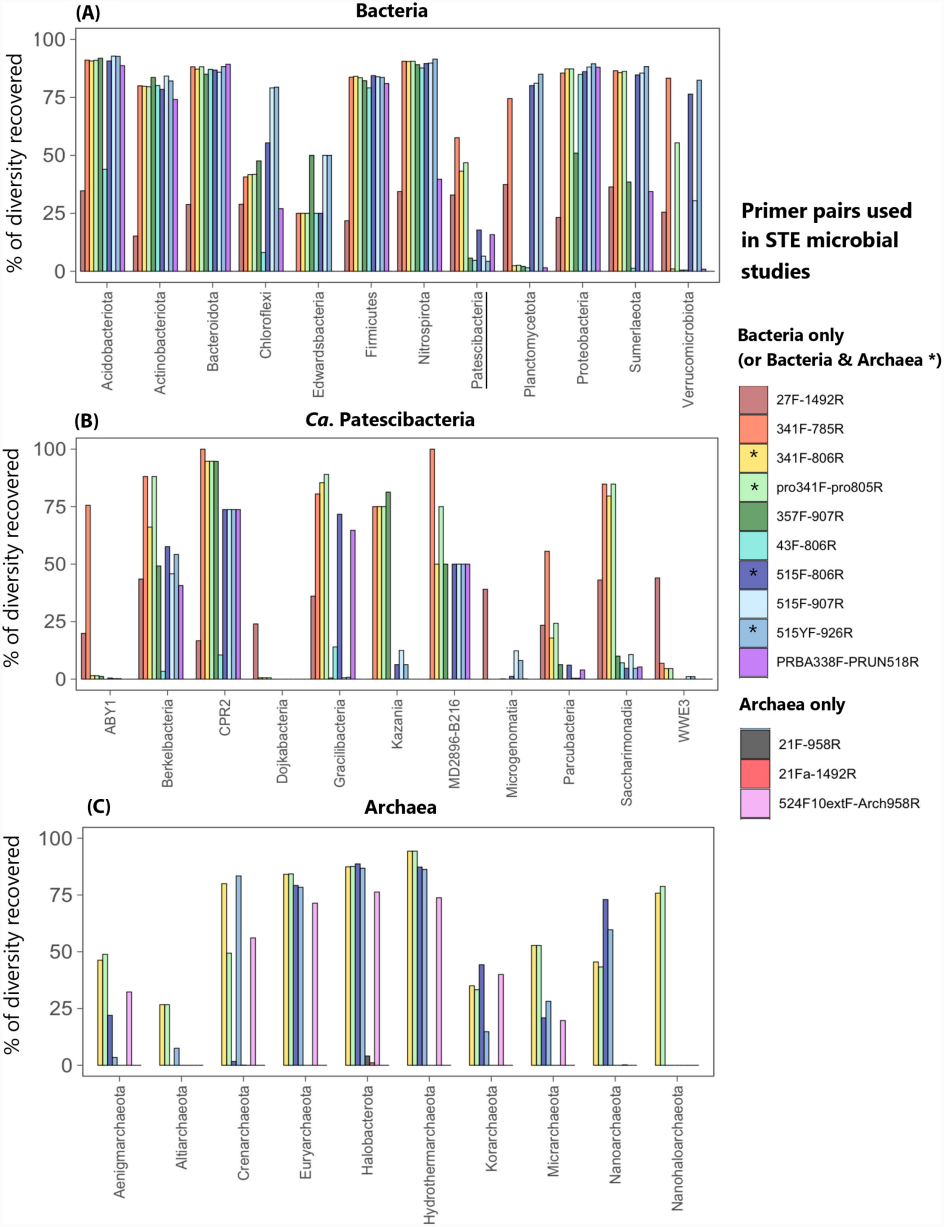
Differential diversity coverage among the 16S rRNA gene primer pairs used in studies on subterranean estuaries (STE). The primers shown include those used in the studies included in Tables S1 and S2 (Supporting Information) to characterize prokaryotic diversity, which represent primers targeting only Archaea, only Bacteria or both (indicated by an asterisk). The different panels represent the diversity coverage of some of **(A)** the main bacterial phyla found in groundwaters, **(B)** the different phyla within the superphylum *Ca*. Patescibacteria (underlined in panel A) and **(C)** the main archaeal phyla known to be present in groundwater. The diversity coverage was tested *in silico* using the online tool TestPrime (Klindworth *et al*. 2013), by comparing the sequences captured by each primer pair against the SILVA taxonomic database, allowing for zero mismatches (i.e. no differences between the compared sequences). Primer sequences are indicated in Table S2 (Supporting Information).

**Figure 4.**
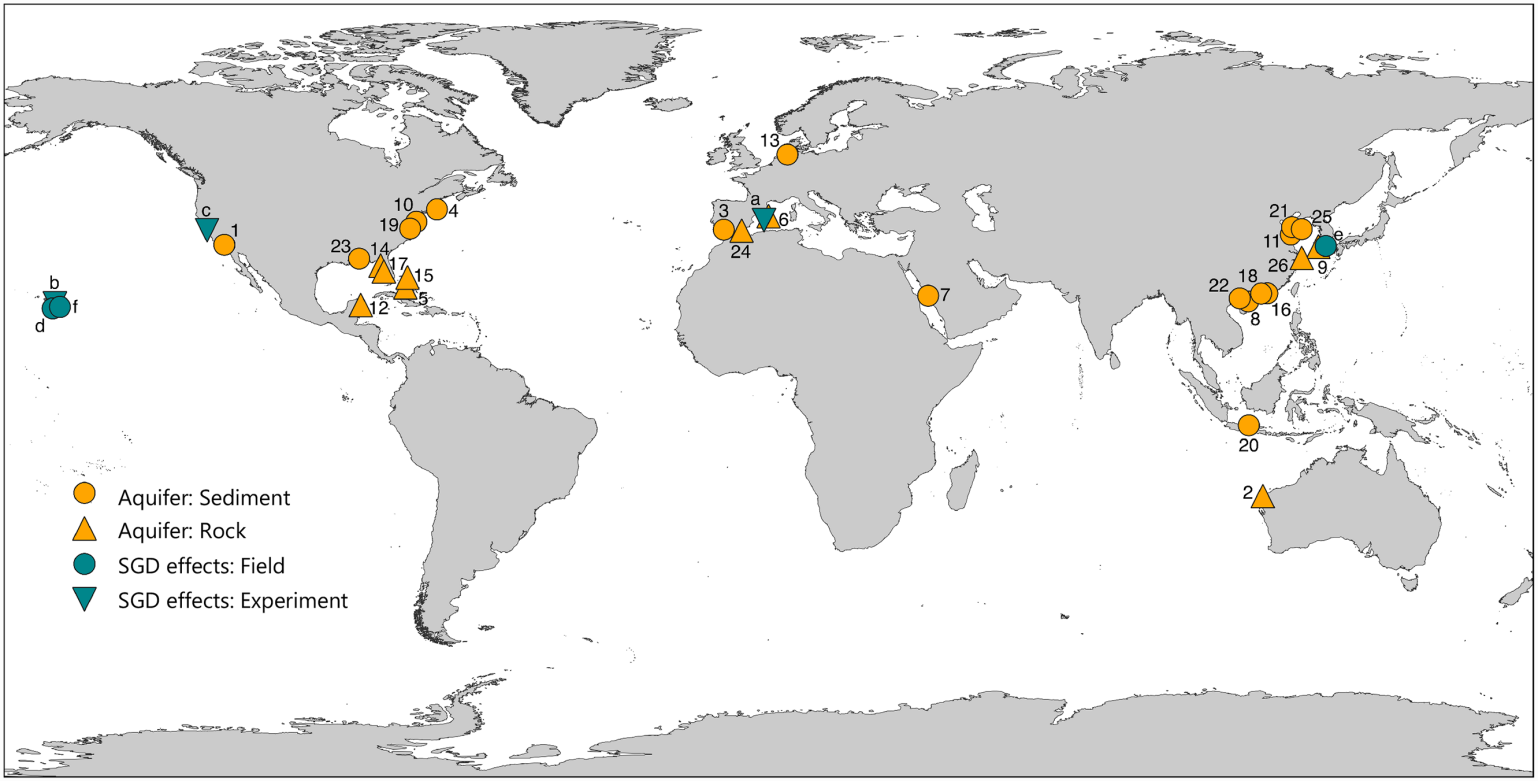
Location of the studies assessing natural prokaryotic communities in coastal aquifers connected to the sea ('Aquifer' studies, orange symbols) and the effects of SGD on natural marine bacterioplankton communities ('SGD effects' studies, turquoise symbols). Microbial investigations in coastal aquifers are distinguished according to the typology of the aquifer (unconsolidated sediments vs carbonate or volcanic rocks; see Table S1, Supporting Information). Experimental and field studies of SGD effects on marine microbial communities are also distinguished (see Table S3, Supporting Information). Numbers correspond to the studied sites reported in Table S1 (Supporting Information), and letters correspond to the studies on SGD effects reported in Table S3 (Supporting Information).

The study of groundwater microorganisms has evolved progressively together with the development of taxa identification techniques (see Griebler and Lueders 2009 for an excellent review on groundwater prokaryotic diversity in inland aquifers since the earliest investigations). A compilation of studies examining microbial communities in coastal aquifers (Table S1, Supporting Information) evidences a clear technical shift moving from approaches such as clone libraries, quantitative polymerase chain reaction (qPCR) or fingerprinting of taxonomic or functional marker genes, to high-throughput sequencing (HTS) technologies such as 454 or Illumina (Logares *et al*. 2012; see Appendices 1 and 2 for definitions of microbiology concepts and methodologies). Currently, most studies on coastal aquifer microbiota have been based on the high-throughput amplicon sequencing of the 16S rRNA gene (Table S1, Supporting Information), which allows a much more detailed characterization of microbial communities than traditional approaches such as clone libraries or fingerprinting techniques. However, this technique may be limited for the description of groundwater microbiota because it relies on the PCR amplification of particular genes (e.g. the 16S rRNA gene) through the use of primers, which are designed based on the sequences available in public databases. Therefore, diversity recovery through primer-based approaches can be biased against taxa that are less represented in public sequence catalogs, such as those from aquifers (and coastal aquifers in particular). In addition, primer pairs targeting different regions of the 16S rRNA gene can be biased against different taxa (Klindworth *et al*. 2013), preventing the direct comparison between studies using different primer pairs. Table S1 (Supporting Information) actually highlights a lack of consensus on primer usage, since up to 13 different 16S rRNA gene primer pairs have been used among the 25 studies describing groundwater prokaryotic diversity in coastal aquifers (Tables S1 and S2, Supporting Information). These primer pairs differ in their coverage of some of the main phyla detected in groundwater (Fig. 3A). Moreover, most fail to capture novel groundwater bacterial lineages discovered by studies applying primer-free approaches (Fig. 3A–C), such as the superphylum *Candidatus* (*Ca*.) Patescibacteria (also known as candidate phyla radiation, CPR), which includes a high diversity of small uncultivated bacterial groups (Brown *et al*. 2015; Luef *et al*. 2015; Anantharaman *et al*. 2016; Castelle *et al*. 2018; He *et al*. 2021; Tian *et al*. 2020; see the section 'Ultrasmall prokaryotic groups are abundant in groundwater ecosystems'), or archaeal members of the DPANN radiation (Diapherotrites, Parvarchaeota, Aenigmarchaeota, Nanoarchaeota, Nanohaloarchaea), also characterized by small cell sizes and genomes (Ludington *et al*. 2017; Castelle *et al*. 2018; He *et al*. 2021). The discovery of these groups has been possible through techniques such as metagenomics and metatranscriptomics that are not biased by the performance of primers (Appendix 2), but so far none of the microbial studies in subterranean estuaries has applied these techniques (Table S1, Supporting Information). Hence, despite the global distribution of the investigations on coastal aquifers conducted so far (Fig. 4), the unknown accuracy of the existing primers to recover groundwater diversity, together with the lack of consensus on which primers to use, precludes generalizations and presumably renders a biased view of groundwater microbial inhabitants in coastal areas.

### Ultrasmall prokaryotic groups are abundant in groundwater ecosystems

Another peculiarity of groundwater microbial communities is that they can shelter minuscule prokaryotes (Brown *et al*. 2015; Luef *et al*. 2015; Proctor *et al*. 2018; Herrmann *et al*. 2019) that may not be captured by current sampling strategies. Prokaryote biomass for DNA extraction or microscopic visualization is often collected onto 0.2-µm-pore-size filters (Table S1, Supporting Information) and these cells can be smaller than this pore size (Nakai 2020). The few groundwater studies (all conducted in inland aquifers) that used sequential filtration of water through 0.2- and 0.1-µm-pore-size filters unveiled that the fraction of small cells passing through the 0.2-µm filters is often dominated by members of the superphylum *Ca*. Patescibacteria (Miyoshi, Iwatsuki and Naganuma 2005; Herrmann *et al*. 2019; He *et al*. 2021) or by the small Archaea belonging to the DPANN radiation (Castelle *et al*. 2018; He *et al*. 2021). The reconstruction of individual genomes from metagenomes (MAGs) or from individually sorted cells (SAGs; Appendix 2) has yielded insight into the metabolic potential of these uncultivated groundwater groups, unveiling unusually reduced genomes and gaps in core metabolic potential that are typical of symbionts (Brown *et al*. 2015; Luef *et al*. 2015; Youssef *et al*. 2015; Anantharaman *et al*. 2016; Castelle *et al*. 2018; Probst *et al*. 2018; He *et al*. 2021). Members of *Ca*. Patescibacteria have been shown to be active in groundwater (Taubert *et al*. 2019) and seem to prefer a planktonic lifestyle (Lazar *et al*. 2019), although their detection in larger planktonic size fractions (e.g. >2 µm) has been interpreted as evidence of episymbiosis (i.e. surface attachment to other microorganisms; He *et al*.^2021^). A recent study, however, claims that *Ca*. Patescibacteria and DPANN genomic features may not be indicative of symbiotic lifestyles but rather of an ancestral absence of electron transport chains, and thus that they rely on fermentative metabolisms (Beam *et al*. 2020). In any case, despite their apparent importance in the subsurface, the relevance of these ultrasmall prokaryotes in coastal aquifers remains unknown due to the previously mentioned primer limitations for capturing these taxonomic groups (Fig. 3), and the lack of studies exploring the diversity within the ultrasmall planktonic fraction (<0.2 µm) in subterranean estuaries (Table S1, Supporting Information; see the section ‘Microbial abundance, diversity and environmental drivers along subterranean estuaries').

### Miniaturized inactive or dormant taxa may be abundant in groundwater

**Figure 5.**
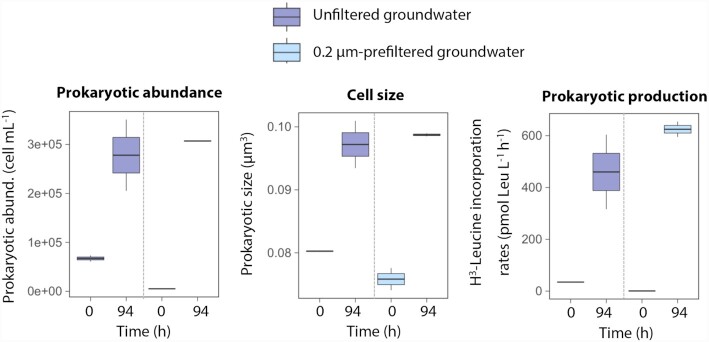
Coastal aquifers hide miniaturized inactive taxa that can reactivate quickly upon changes in conditions. Increase in prokaryote abundances, average cell size and prokaryotic heterotrophic activity (prokaryotic production, measured as the incorporation of radioactive leucine) after an ∼4-day incubation (*in situ* temperature, dark conditions, no nutrient or carbon addition) of groundwater collected from a coastal alluvial aquifer (Argentona, western Mediterranean; see Folch *et al*. 2020). Groundwater samples representing unfiltered (dark blue) and filtered through a 0.2-µm-pore-size filter (light blue) are distinguished. Prokaryotic abundance and cell size were estimated by flow cytometry (Gasol and del Giorgio 2000), and prokaryotic production was estimated measuring the incorporation of radiolabeled ^3^H-leucine (Kirchman *et al*. 1985; Smith and Azam 1992). Note that bacteria passing through the 0.2-µm filter became larger in size and increased their abundances and activity, suggesting that they were not true ultra-microbacteria, but rather miniaturized cells due to the prevalent conditions of organic carbon limitation.

Prokaryotes can also miniaturize in response to stress or starvation conditions (Velimirov 2001) becoming inactive or dormant (i.e. in a reversible state of low metabolic activity; Lennon and Jones 2011). Due to the typical scarcity of freshly produced carbon in subsurface ecosystems (Hofmann and Griebler 2018), groundwater microbes have probably evolved diverse strategies to persist in resting or dormant states until more favorable conditions occur. Given that an important fraction of the cells in aquifers are believed to be inactive (Griebler and Lueders 2009), it is possible that a fraction of the ultrasmall cell pool represents miniaturized dormant taxa rather than obligate ultra-microbacteria (*sensu* Nakai 2020). In support of this, the exposure of bacterial groundwater isolates to the oligotrophic conditions typical of pristine aquifers caused a significant cell size reduction (Herrmann *et al*. 2019). Also, we have observed that the incubation of 0.2-µm prefiltered groundwater from a coastal Mediterranean aquifer results in pronounced increases in the abundance, cell size and heterotrophic production of the bacteria passing through the 0.2-µm filter (Fig. 5), indicating that some cells had been miniaturized *in situ* (and were presumably dormant) likely due to the prevailing carbon scarcity.

Such miniaturized cells could comprise a microbial 'seed bank' (i.e. a reservoir of dormant bacteria that can reactivate upon changes in conditions; Lennon and Jones 2011). In general, though, little is known about the relevance and extent of dormancy in groundwater systems. A recent experimental study suggested that bacteria in oligotrophic aquifers can be locally co-limited by organic carbon and phosphorous, but that they can reactivate and reach high bacterial abundances when amended with different organic carbon sources and nutrients (Hofmann and Griebler 2018), or with soil dissolved organic matter (DOM) richer in labile compounds (Hofmann *et al*. 2020). Studies in deep marine sediments, where microbes face increasing starvation with depth, have shown that dormant endospore-forming cells can outnumber actively growing cells (Wörmer *et al*. 2019) and that most microorganisms appear to be surviving instead of growing (Bradley, Amend and LaRowe 2019). Thus, rather than growth, maintenance mechanisms might be key to thrive in such oligotrophic habitats. Finally, environmental stresses such as salinity changes also appear to trigger dormancy in bacteria (Sachidanandham and Yew-Hoong Gin 2009; Aanderud *et al*. 2016), so this mechanism might allow repeated activation and inactivation of prokaryotes exposed to changing conditions caused by the mixing of seawater and groundwater in coastal aquifers.

Groundwater microbial communities might also include 'allochthonous' microorganisms transported from external sources that are hydraulically connected to the aquifer (e.g. soils, rivers, lakes; Hubalek *et al*. 2016; Graham *et al*. 2017; Meier *et al*. 2017; Herrmann *et al*. 2019, Fillinger *et al*. 2021). Although some of these transported microorganisms may comprise a travelling groundwater 'seed bank', being able to colonize different habitats within groundwater ecosystems (such as sediments or rock surfaces; Griebler *et al*. 2002; Griebler, Malard and Lefébure 2014; Fillinger *et al*. 2019b; Herrmann *et al*. 2019; see the section 'Groundwater biomass and activity is concentrated on aquifer solids'), others might just be allochthonous maladapted taxa able to persist inactively (or dead) during transit in groundwater, with no influence in this subsurface ecosystem.

The presence of inactive or allochthonous taxa may be of special relevance in coastal aquifers, where communities may be continuously subjected to a bidirectional inoculation of taxa from the marine and fresh groundwater endmembers (Adyasari *et al*. 2019). However, whether these transported microorganisms tolerate or inactivate upon such wide ranges of conditions remains an open question, mostly because all studies assessing microbial diversity have been based on sequencing of the 16S rRNA gene (Table S1, Supporting Information), which does not allow distinguishing between the active, inactive and even dead components of communities. The recent finding that extracellular or 'relic' DNA can persist for long periods in soils and sediments (Carini *et al*. 2016) further implies that some of the detected taxa might simply be remnants of formerly active communities, and the very small proportion of the total microbial biomass (0.02–6.36%) found to be active in a coastal alluvial aquifer along a two-year period (Velasco Ayuso *et al*. 2010) supports that only a small fraction of the detected diversity may be locally functioning in these coastal systems. Distinguishing between the active and the inactive (dormant or dead) groundwater microbial community members is therefore essential, because the inactive pools of taxa may mask spatial or temporal patterns in taxonomic composition, obscuring our comprehension of the observed linkages between the environment, the taxonomic structure of communities, and their functions (Niño-García, Ruiz-González and del Giorgio 2016; Wisnoski *et al*. 2020).

### Groundwater biomass and activity is concentrated on aquifer solids

Compared with surface aquatic ecosystems, aquifers tend to have much higher solid–water ratios and slower flow velocities (usually on the order of millimeters to meters per day), which allows for an intense interaction between groundwater and solids that influences groundwater chemistry and surface characteristics of the geological matrix (Moore 2010; Tovar-Sánchez *et al*. 2014). This physical structure of aquifers challenges the study and the sampling of aquifer microbiota, as prokaryotes may not occur only as free-living planktonic cells but also attached onto solid aquifer surfaces as individual cells or aggregates (Smith *et al*. 2018). Actually, most groundwater microbial biomass and activity seems to be concentrated on solid surfaces (Griebler *et al*. 2002; Smith *et al*. 2018), often showing a highly patchy distribution at the microscale due to the microniches than can be established within pores and cavities (Goldscheider, Hunkeler and Rossi 2006; Schmidt, Cuthbert and Schwientek 2017; Smith *et al*. 2018).

Not many studies have compared the free-living and the attached microbial groundwater components. In inland aquifers, these investigations have shown that microbial communities adhered to karst sediments display higher activities than the suspended ones, likely due to a better access to organic carbon and nutrients (Wilhartitz *et al*. 2009). The ratio of attached versus free-living bacteria was shown to increase pronouncedly as oligotrophic conditions increased, ranging from ∼50:1 in a contaminated site to >1600:1 in pristine groundwater (Griebler *et al*. 2002). Attached microbial communities differ notably from the planktonic ones in their taxonomic composition, as has been shown by the direct sampling of microorganisms from aquifer rocks and groundwater (Herrmann *et al*. 2017; Meier *et al*. 2017; Lazar *et al*. 2019), or by incubation of sterilized sediments within wells (Zhou, Kellermann and Griebler 2012; Flynn *et al*. 2013) and in experimental microcosms or mesocosms (Longnecker and Kujawinski 2013; Fillinger *et al*. 2019b). Results from these studies show that groundwater transports taxa able to colonize sediments (Fillinger *et al*. 2019b) and that attached communities are more stable over time than planktonic microorganisms (Zhou, Kellermann and Griebler 2012), but more responsive to the presence of bacterial grazers (Longnecker and Kujawinski 2013).

No study on coastal aquifers has directly compared groundwater and sediment microbial communities (Table S1, Supporting Information). However, prokaryotes living on sand grains and in saline porewater were found to differ at fine taxonomic levels (Gobet *et al*. 2012), and communities from two planktonic size fractions (0.2 and 0.45 µm, which may include small suspended particles) differed taxonomically in a subterranean estuary (Chen *et al*. 2019b). Hence, targeting only the planktonic or the sediment-attached compartment may lead to a rather incomplete view of microbial ecology in inland or coastal groundwater. Given the relevance of hydrologic boundaries and flow paths for the assembly and functioning of the biofilm communities in inland shallow aquifers (Smith *et al*. 2018), it is likely that subterranean estuaries are also characterized by a highly complex spatiotemporal structuring of the sediment-attached microbial component.

## MICROBIAL ECOLOGY AT THE TERRESTRIAL–MARINE INTERFACE CONNECTED BY GROUNDWATER

Environmental transitions or ecotones are relevant landscape components. Due to the mixing of resources and communities, these sites are often hotspots of biogeochemical activity, where abrupt physicochemical changes or gradients determine large variations in microbial community composition and activity (e.g. McClain *et al*. 2003; Stegen *et al*. 2016). Subterranean estuaries (Box 1), which are ubiquitous along coastlines, represent ideal systems to study microbial adaptations to the abrupt environmental transitions created by the mixing of groundwater and seawater, but they have been largely understudied compared with surface waters, estuaries or inland aquifers. In this section, we review the current knowledge on microbial communities from coastal aquifers based on the available studies (Table S1, Supporting Information; Fig. 4), highlighting what we have learned about the microbial diversity, drivers and functioning of these unexplored ecosystems. We also provide an overview of the few studies exploring the effects of SGD on coastal bacterioplankton communities and the potential ecological implications (Table S3, Supporting Information; Fig. 4).

### Microbial abundance, diversity and environmental drivers along subterranean estuaries

Despite the recent rise in the number of microbial studies focusing on aquifers connected to the sea (Table S1, Supporting Information; Fig. 4), little is known regarding the abundance, diversity and the roles of the microorganisms inhabiting these systems. Only a few studies have quantified prokaryotic cell abundances or bulk heterotrophic production in subterranean estuaries (Table S1, Supporting Information), even though this is something that is routinely done in microbial investigations on surface waters. Applying epifluorescence microscopy to porewater samples in a sandy beach aquifer, Santoro *et al*. (2008) reported up to 1.4 × 10^6^ cells mL^−1^. Higher maximum abundances were observed in a coastal alluvial aquifer that is well connected to the surface, where large seasonal variations in cell numbers (range 2 × 10^5^ to >6 × 10^7^ cells mL^−1^) were related to changes in temperature and nutrient concentrations (Velasco Ayuso *et al*. 2009a,b). These values fall all in the upper range of those typically found in pristine inland groundwater (10^2^–10^6^ cells mL^−^^1^; Griebler and Lueders 2009), but the scarcity of data prevents any accurate comparison. In sediments, up to 2.6 × 10^8^ cells cm^−3^ were measured (Beck *et al*. 2017), implying that likewise in inland aquifers, coastal sediments harbor higher prokaryotic biomass than ground- or porewater (see the section 'Groundwater biomass and activity is concentrated on aquifer solids').

In terms of microbial community composition, the recent application of high-throughput sequencing technologies (Table S1, Supporting Information) has shown that subterranean estuaries hide heterogeneous and diverse prokaryotic communities varying pronouncedly at small vertical and horizontal scales (e.g. Héry *et al*. 2014; Unno *et al*. 2015; Ye *et al*. 2016; Beck *et al*. 2017; Adyasari *et al*. 2019; Chen *et al*. 2019a; Hong *et al*. 2019; Adyasari *et al*. 2020; Chen *et al*. 2020b; Sola, Vargas-García and Vallejos 2020). Such studies have generally reported a dominance of the bacterial phyla Proteobacteria (mostly Gamma- and Alphaproteobacteria), Bacteroidetes and/or Actinobacteria, with other phyla like Planctomycetes, Chloroflexi or the endospore-forming Firmicutes being locally important depending on the environmental conditions (Héry *et al*. 2014; McAllister *et al*. 2015; Unno *et al*. 2015; Ye *et al*. 2016; Sang *et al*. 2018, 2019; Chen *et al*. 2019a; Hong *et al*. 2019; Zhang *et al*. 2021). Taxa belonging to the ultrasmall *Ca*. Patescibacteria have rarely been observed, often comprising <2% of the 16S rRNA sequences (Héry *et al*. 2014; Unno *et al*. 2015; Ye *et al*. 2016; Chen *et al*. 2019a), but locally reaching up to 10% (Adyasari *et al*. 2020). These abundances might be underestimates due to the previously mentioned primer biases for capturing these novel groups (see the section 'Novel groundwater microbial taxa can be missed by current sequencing technologies'; Fig. 3) and/or to the loss of ultrasmall cells during 0.2 µm filtration (Table S1, Supporting Information; see the section 'Ultrasmall prokaryotic groups are abundant in groundwater ecosystems').

Archaea are generally less abundant than bacteria, but they harbor functional groups such as methanogens, ammonia oxidizers or nitrifiers that may play key roles in aquifer biogeochemistry (Santoro *et al*. 2008; Davis and Garey 2018; Adyasari *et al*. 2020; see the section 'Microbial processes within subterranean estuaries'). Archaea often comprise <1% of total prokaryotic sequences in the planktonic compartment (Unno *et al*. 2015; Adyasari *et al*. 2019) but they can reach higher abundances (5% up to 50%) under specific conditions in both groundwater (Sang *et al*. 2018; Adyasari *et al*. 2020) or sediments (Missimer *et al*. 2014; Hong *et al*. 2019). Euryarchaeota, Chrenarchaeota, Thaumarchaeota and *Ca*. Woesearchaeota are generally the dominant archaeal phyla (Rogers and Casciotti 2010; Missimer *et al*. 2014; Unno *et al*. 2015; Davis and Garey 2018; Sang *et al*. 2018; Hong *et al*. 2019; Adyasari *et al*. 2020) but phyla such as *Ca*. Bathyarchaeota and *Ca*. Parvarchaeota (the latter belonging to the DPANN radiation) have also been observed (Chen *et al*. 2019b; Hong *et al*. 2019).

Temperature, salinity, pH, dissolved oxygen and nutrient concentration, redox conditions and DOM quality have commonly been identified as potential environmental drivers of the observed compositional variations in prokaryotic communities from coastal aquifers (McAllister *et al*. 2015; Beck *et al*. 2017; Davis and Garey 2018; Jiao *et al*. 2018; Adyasari *et al*. 2019, 2020; Chen *et al*. 2019a; Hong *et al*. 2019; Jiang *et al*. 2020; Sola, Vargas-García and Vallejos 2020). Hydrological conditions and connectivity with surrounding ecosystems, although less commonly studied, also appear as relevant drivers of the distribution of communities in these transition zones. For example, changes in microbial abundances, activity or composition have been reported in coastal aquifers driven by dynamic changes in environmental gradients due to tides or waves (Santoro *et al*. 2008; McAllister *et al*. 2015), seasonal changes in groundwater level (Menning *et al*. 2018; Chen *et al*. 2020a) or varying degrees of connectivity with the surface or coastal seawater (Velasco Ayuso *et al*. 2009a,b, 2010, 2011; Adyasari *et al*. 2019).

The reported changes in microbial community structure cannot always be predicted from the mixing of freshwater and seawater, since the specific conditions in the mixing zone promote the establishment of unique and diverse populations of taxa that are rare elsewhere (Héry *et al*. 2014; Hong *et al*. 2019). This is in accordance with experimental studies showing that the mixing of freshwater and marine bacterial assemblages can promote the growth of initially rare taxa (Shen, Jürgens and Beier 2018; Rocca *et al*. 2020). As the activation of rare taxa can impact relevant ecosystem processes (Sjöstedt *et al*. 2012; Aanderud *et al*. 2015; Stegen *et al*. 2016), such hotspots of microbial specialists along subterranean estuaries could have strong implications in aquifer biogeochemistry. However, most of the microbial investigations in subterranean estuaries have been restricted to a single sampling time or have low spatial resolution (Table S1, Supporting Information), so we lack a comprehensive understanding of the hydrologic and environmental drivers of these communities over time and space.

### Microbial processes within subterranean estuaries

**Figure 6.**
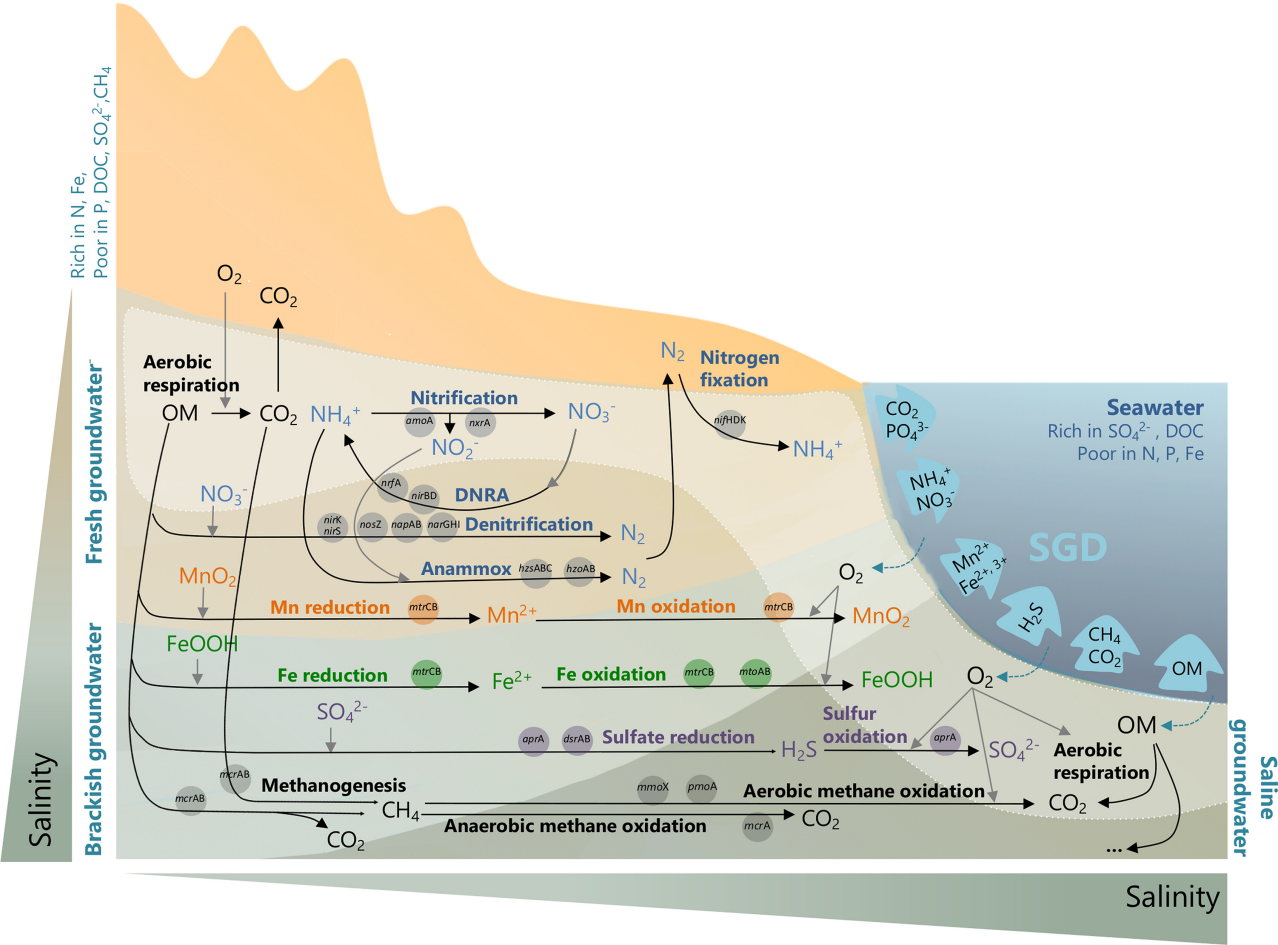
Simplified representation of main biogeochemical processes mediated by prokaryotes in subterranean estuaries of importance for SGD chemical fluxes, and key functional marker genes. The different color fonts indicate metabolic pathways involved in the cycling of carbon (black), nitrogen (blue), manganese (orange), iron (green) and sulfur (purple). The oxidation of the reduced products deriving from these respiratory processes (i.e. NH_4_^+^, Mn^2+^, Fe^2+^, H_2_S) is often coupled to CO_2_ fixation by chemolithoautotrophs. Gray arrows denote electron acceptors; black solid arrows represent the transformations of chemicals. Marker genes used in groundwater studies to identify microbial functional groups involved in each process are indicated in bubbles (and are defined in Table 1). The most common products of biotic transformations in the subterranean estuary that might be delivered to the ocean through SGD are indicated inside the light blue arrows. This is an abstraction of the system, assuming that the freshwater–saltwater mixing zone behaves as a redox boundary between oxic fresh groundwater and saline groundwater of lower oxygen content, and that the circulation of oxygenated seawater through the surface layer of marine sediments (lighter area) also leads to a comparable sequence of transformations established along a depth profile (indicated as '…'). DNRA, dissimilatory nitrate reduction to ammonium; anammox, anaerobic ammonium oxidation; OM, organic matter.

Microbial activity in subterranean estuaries is mainly fueled by the degradation of organic matter or the oxidation of inorganic compounds supplied by fresh groundwater and/or infiltrated seawater (Seibert *et al*. 2020; Fig. 6). For heterotrophic microorganisms, the principal pathways of organic matter degradation vary depending on the availability of electron acceptors, and include aerobic respiration, denitrification, manganese reduction, iron reduction, sulfate reduction and methanogenesis (Canfield and Thamdrup 2009). These pathways usually occur in a predictable sequence along a groundwater flow path, which results in freshwater aquifers usually having a characteristic spatial zonation of redox conditions and active microbial populations (Hunter, Wang and Van Cappellen 1998). In coastal aquifers, this metabolic zonation may be much more dynamic given that the availability of organic matter, oxygen and other electron acceptors and donors can change over time due to the dynamic mixing of fresh and marine waters and the complex interaction of terrestrial and marine forces (Slomp and Van Cappellen 2004; McAllister *et al*. 2015; Robinson *et al*. 2018; Fig. 6). Hence, once the available oxygen supplied by fresh groundwater or seawater is consumed by aerobic microorganisms, other electron acceptors that are differentially delivered by fresh- or marine water will be used for anaerobic respiration by denitrifiers, manganese- and iron-reducers, and finally by sulfate reducers and methanogens (McAllister *et al*. 2015; Beck *et al*. 2017; Hong *et al*. 2019; Montiel *et al*. 2019). In turn, the reduced products of these reactions can be oxidized by other microorganisms through processes such as anammox (anaerobic ammonium oxidation), nitrification, and iron, sulfur or methane oxidation, which are often coupled to CO_2_ fixation by chemolithoautotrophs (Santoro, Boehm and Francis 2006; Santoro *et al*. 2008; McAllister *et al*. 2015; Fig. 6). Although accumulating evidence from inland aquifers suggests that chemolithoautotrophy can be more important than previously believed in subsurface ecosystems (Alfreider, Schimer and Vogt 2012; Kellermann *et al*. 2012; Herrmann *et al*. 2015; Hutchins *et al*. 2016; Jewell *et al*. 2016; Brankovits *et al*. 2017), the relative contribution of autotrophic metabolisms to total carbon cycling is currently unknown in subterranean estuaries.

The physicochemical conditions imposed by the mixing between fresh groundwater and marine seawater in the subterranean estuary lead to a dynamic and heterogeneous distribution of functional capacities and metabolic rates. Salinity, for example, is known to impose a strong filter for microbial communities, as large changes in microbial community composition are always found along salinity gradients (Lozupone and Knight 2005). In a comprehensive review on the microbial nitrogen cycling in subterranean estuaries, Santoro (2010) suggested that increases in salinity may favor metabolisms such as DNRA, sulfate reduction and carbon remineralization while decreasing denitrification, nitrification and methanogenesis. Both anammox and denitrification potential rates were shown to decrease with increasing salinity in low permeable intertidal sediments (Jiao *et al*. 2018). Also, higher rates of methanogenesis within subterranean estuaries have been observed in fresher or brackish portions than in sulfate-rich saltwater sites (Brankovits *et al*. 2018; Pain, Martin and Young 2019), given that methanogens may be outcompeted for chemical substrates by sulfate-reducers (Whiticar 1999).

Besides salinity, many other factors such as redox conditions can differentially affect the distribution of microbial functional groups. Slomp and Van Cappellen (2004) classified subterranean estuaries based on the redox state of the fresh and saline groundwater that mix in the coastal zone, which has been proposed as a useful framework for predicting biogeochemical cycles in different subterranean estuaries (Santoro 2010). However, reality is extremely much more complex because there can be overlap in redox zones (Canfield and Thamdrup 2009) and a large spatiotemporal heterogeneity in this metabolic zonation at the microscale; for instance, discrete microenvironments and microbial communities can be established within interstitial pores (Schmidt, Cuthbert and Schwientek 2017; Smith *et al*. 2018), and respiration processes can be spatially restricted to the fringes of contaminant plumes due to depletion of electron acceptors in the plume core (Meckenstock *et al*. 2015; Pilloni *et al*. 2019). In any case, in order to be able to predict the distribution of different microbial groups and their functions, we need to gain insight into their environmental tolerances, preferences and metabolic capacities.

### Dynamics of microbial functional groups in coastal aquifers

The recent application of approaches such as metagenomics and metatranscriptomics, or stable isotope probing (SIP), has yielded much insight into the metabolic potential of groundwater microbial communities from inland aquifers (see the section 'Improving the description of microbial diversity and function in coastal aquifers'). On the contrary, most recent studies on subterranean estuaries have exclusively focused on the taxonomic composition of communities (Table S1, Supporting Information), which does not provide information on their metabolisms or activity rates. Some of these studies have attempted to predict microbial function based on taxonomic identity (Table S1, Supporting Information), unveiling a high diversity and highly heterogeneous distributions of potential microbial metabolisms (Davis and Garey 2018; Hong *et al*. 2019; Adyasari *et al*. 2020) and highlighting the importance of freshwater–saltwater transition zones as potential hotspots of functional diversity (Santoro, Boehm and Francis 2006; Hong *et al*. 2019). Although these metabolic predictions may be inaccurate or biased because they depend on the sequenced genomes available (Sun, Jones and Fodor 2020), the identification of particular taxa with known metabolisms might provide useful clues into the processes that could be operating in a given area (Montiel *et al*. 2019).

Other studies in subterranean estuaries have directly tracked functional microbial groups using primer-based approaches such as clone libraries or quantitative PCR (qPCR). These have reported pronounced spatial and temporal variations in the abundance or diversity of genes such as the ammonia monooxygenase gene (*amo*A) involved in the aerobic oxidation of ammonia (Santoro *et al*. 2008; Rogers and Casciotti 2010; Beck *et al*. 2017), nitrate reductase encoding genes (*nir*K and *nir*S) involved in denitrification (Santoro, Boehm and Francis 2006; Jiao *et al*. 2018) or genes involved in sulfate reduction (*apr*A) (Beck *et al*. 2017) (Fig. 6; Table 1). Metabolic biomarkers such as ladderane phospholipids or specific 16S rRNA gene primers have also been used to quantify anammox bacteria in subterranean estuaries (Sáenz *et al*. 2012; Jiao *et al*. 2018) as well as respiratory quinone composition for distinguishing certain metabolisms such as aerobic methanotrophy (Brankovits *et al*. 2017).

**Table 1.** Major metabolic pathways and some of the relevant marker genes targeted in groundwater studies.

Metabolic pathway	Gene	Protein	Groundwater studies
Aerobic respiration	*cyo*A	Cytochrome bo(3) ubiquinol oxidase subunit 2	a
	*cyd*A	Cytochrome bd-I ubiquinol oxidase subunit 1	a
	*cco*N	Cytochrome c oxidase	a,b
	*cox*A	Cytochrome c oxidase subunit I	a
Autotrophic carbon fixation	*cbb*L	Ribulose bisphosphate carboxylase large chain	b,c,f
	*cbb*M	Ribulose bisphosphate carboxylase	c,f
	*cdh*A	Acetyl-CoA decarbonylase/synthase complex subunit α	b
	*cdh*B	Acetyl-CoA decarbonylase/synthase complex subunit ε	b
Nitrification	*amo*A	Ammonium monooxygenase subunit A	a**,d,e,i,j,k**
	*nxr*A	Nitrite oxidoreductase α subunit	a,**i**
Anammox	*hzs*A	Hydrazine synthase subunit A	b
	*hzs*B	Hydrazine synthase subunit B	b
	*hzs*C	Hydrazine synthase subunit C	b
	*hzo*A	Hydrazine oxidoreductase A	b
	*hzo*B	Hydrazine oxidoreductase B	b
Denitrification	*nir*K	Copper-containing nitrite reductase	a,b,**h**,**i,k**
	*nir*S	Nitrite reductase	a,b,**h,i**
	*nos*Z	Nitrous-oxide reductase	a,b,**i,k**
	*nap*A	Periplasmic nitrate reductase	a,b,**i**
	*nap*B	Periplasmic nitrate reductase, electron transfer subunit	a,b,**i**
	*nar*G	Respiratory nitrate reductase 1 α chain	a,b,**i**
	*nar*H	Respiratory nitrate reductase 1 β chain	a,b,**i**
	*nar*I	Respiratory nitrate reductase 1 γ chain	b,**i**
DNRA	*nrf*A	Nitrite reductase (formate dependent)	**i,k**
	*nir*B	Nitrite reductase (NADH-dependent) large subunit	**i**
	*nir*D	Nitrite reductase (NADH-dependent) small subunit	**i**
Nitrogen fixation	*nif*D	Nitrogenase molybdenum-iron protein α chain	a,**i**
	*nif*H	Nitrogenase iron protein	a,**i,k**
	*nif*K	Nitrogenase molybdenum-iron protein β chain	a,**i**
Iron oxidation	*mto*A	Decaheme c-type cytochrome	b
	*mto*B	Decaheme-associated outer membrane protein	b
Iron/manganese oxidation/reduction	*mtr*B	Decaheme-associated outer membrane protein	a
	*mtr*C	Decaheme c-type cytochrome	a
Sulfur oxidation/reduction	*apr*A	Dissimilatory adenosine-5′-phosphosulfate reductase	a,b,**e**
Sulfate reduction	*dsr*A	Sulfite reductase α subunit	a,b,**k,l**
	*dsr*B	Sulfite reductase β subunit	a,b
Methanotrophy (aerobic)	*pmo*A	Membrane-bound particulate methane monooxygenase	g,m
	*mmo*X	Soluble methane monooxygenase	g,**k**
Methanotrophy (anaerobic)/methanogenesis	*mcr*A	Methyl-coenzyme M reductase α subunit	**k,l**,m

Letters indicate examples of groundwater studies where the functional marker genes shown in Fig. 6 have been identified (or predicted from taxonomy as in '**i**', '**k**' and '**l**'), plus some genes indicative of aerobic respiration and autotrophic carbon fixation. Studies performed in **subterranean aquifers** are indicated in **bold** (see also Table S1, Supporting Information), but examples of inland aquifer investigations where other marker genes have been targeted are also included. Anammox (anaerobic ammonium oxidation), DNRA (dissimilatory nitrate reduction to ammonium), RuBisCo (ribulose-1,5-bisphosphate carboxylase/oxygenase). (a) Lavy *et al*. 2019; (b) Jewell *et al*. 2016; (c) Herrmann *et al*. 2015; **(d)Santoro *et al*.^2008^**; **(e)Beck *et al*.^2017^**; (f) Kellerman *et al*. 2012; (g) Shao *et al*. 2019; **(h)Santoro *et al*.^2006^**; **(i)Adyasari *et al*.^2020^**; **(j)Rogers and Casciotti 2010**; **(k)Hong *et al*.^2019^**; **(l)Sang *et al*.^2018^**; (m) Vigneron *et al*. 2017.

Despite this accumulating knowledge, our understanding of the links between the taxonomic composition of a community and its biochemical outcome is still limited, in part because microbial studies in coastal aquifers have rarely coupled microbial information with actual metabolic rates (Table S1, Supporting Information). For example, the different responses of ammonia-oxidizing bacteria or ammonia-oxidizing archaea to salinity resulted in large shifts in the ratio between the two along a subterranean estuary (Santoro *et al*. 2008), but the implications of this taxonomic shift for the *in situ* nitrification rates remain unknown. Using stable nitrogen tracer (^15^NO_3_^−^) assays, decreases in anammox and denitrification rates were observed with increasing salinity, but these were not accompanied by decreases in the estimated abundances of the responsible microorganisms (Jiao *et al*. 2018). Once again, PCR primer limitations, presence of inactive taxa or different activity rates between taxonomically different groups may be among the reasons explaining this lack of coupling between microbial identity and function. In addition, there may be functional redundancy among groundwater taxa, which implies that a given process could be maintained even if the main microbial players are replaced (Pilloni *et al*. 2019). Given that sequencing techniques have replaced, rather than complemented, the use of traditional bulk activity assays such as estimates of active biomass, heterotrophic production or exoenzymatic activity (Table S1, Supporting Information), the gain in taxonomic resolution has been accompanied by a notable loss of information on the actual functioning of communities and its regulation. As noted by Smith *et al*. (2018), merging traditional activity measurements with novel sequencing technologies may provide the needed multifaceted view of these underexplored communities.

### Microbial control of SGD-derived chemical fluxes

Ultimately, microbial activity, together with other geochemical reactions occurring in the subterranean estuary (e.g. desorption, dissolution, precipitation), will determine the chemical composition of the groundwater flowing through coastal aquifers into the ocean (Moore 1999; Slomp and Van Cappellen 2004; Seibert *et al*. 2020; Fig. 6). Microbial activity in coastal sediments was shown to reduce significantly anthropogenic nitrate through denitrification and DNRA, acting as a natural filter, whereas organic matter mineralization resulted in large amounts of ammonia and dissolved organic nitrogen to the ocean (Montiel *et al*. 2019). Other studies have reported that processes such as methanogenesis and methanotrophy are important sources and sinks of methane in subterranean estuaries, largely determining SGD methane fluxes to the ocean (Schutte *et al*. 2016; Brankovits *et al*. 2018; Pain, Martin and Young 2019), and that the transport of particulate marine matter through the seepage zone can fuel microbial dissolved organic carbon production, resulting in a net release of SGD-driven DOM (Jiang *et al*. 2020). The different seasonal patterns shown by denitrification, DNRA or anammox in a sandy subterranean estuary further illustrate that the multiple microbial processes involved in the cycling of a given element (e.g. nitrogen) may be differentially regulated (Wong *et al*. 2020). Consequently, only a deep understanding of these processes and their natural controls will enable an accurate understanding or prediction of the potential changes in SGD-derived fluxes upon variations in environmental conditions.

### Influence of SGD on marine microbial communities

Since the awareness of the global relevance of SGD in coastal biogeochemical processes, multiple investigations have explored the effects of SGD on marine biota, including macro- and microorganisms (see Lecher and Mackey 2018 for a review). Microbial-related SGD studies have focused mostly on the responses of phytoplankton communities because of their key role as marine primary producers, and have shown that the nutrient inputs or salinity changes associated with SGD often result in variations in the abundances and composition of planktonic communities, promoting the growth of certain groups and sometimes causing eutrophication or harmful algal blooms (Lecher and Mackey 2018; Adolf *et al*. 2019; Taniguchi *et al*. 2019; Chen *et al*. 2020c). Despite the relevance of marine bacteria for the functioning of coastal biogeochemistry (Gasol *et al*. 2008), much less is known about marine bacterioplankton taxonomic and functional responses to SGD, as the few available studies have focused mostly on exploring changes in bulk abundances or activity of bacteria (Table S3, Supporting Information; Fig. 4). Photosynthetic unicellular cyanobacteria such as *Synechococcus* and *Prochlorococcus* are responsible for ∼25% of the primary production globally (Kirchman 2012) and can be important primary producers also in coastal waters (Agawin *et al*. 2003; Scanlan *et al*. 2009). Experimental addition of groundwater to marine communities resulted in fast abundance increases of *Synechococcus* in the Mediterranean Sea (Garcés, Basterretxea and Tovar-Sánchez 2011), but not in other coastal settings (California and Hawaii) where other phytoplankton groups were favored (Chamberlain *et al*. 2014; Lecher *et al*. 2015). These findings agree with experiments reporting variable responses of coastal *Synechococcus* to different nutrient additions (Lekunberri *et al*. 2012), and highlight that microbial responses to SGD will depend largely on the chemical composition of groundwater and specific local and biotic conditions.

On the other hand, heterotrophic bacterioplankton largely determine the flow and fate of carbon in the ocean. Community-level processes such as bacterial respiration or biomass production thus have implications at the ecosystem level (del Giorgio and Williams 2005; Gasol *et al*. 2008), and appear to be affected by SGD (Carlson and Wiegner 2016). The recurrent observation that coastal bacterial respiration rates often exceeds primary production indicates that bacteria rely on allochthonous (i.e. land-derived) dissolved organic carbon (Duarte, Agustí and Vaqué 2004; Duarte and Prairie 2005), but to our knowledge no study has explored how much of this biologically used carbon derives from SGD.

Bacteria are also relevant competitors for inorganic nutrients, accounting for ∼40% of total uptake of inorganic phosphorus, nitrogen and iron (Kirchman 2000). Bacterial ability to outcompete eukaryotic phytoplankton may vary depending on the type of inorganic nutrients and carbon added, and hence the chemical composition of the SGD may determine the biological fate of nutrients or carbon. For example, bacteria outcompeted diatoms in mesocosms with additions of phosphate and nitrate (with or without glucose), but if silicate was added, diatoms competed successfully with bacteria for the uptake of mineral nutrients (Havskum *et al*. 2003). In the study of Garcés *et al*. (2011) only a moderate response in the total number of bacteria upon groundwater addition was reported after 3 days of incubation, probably caused by a better performance of phytoplankton upon the increased availability of silicate. However, marine bacteria are the fastest responders to nutrient inputs (e.g. Lekunberri *et al*. 2012), so SGD-driven effects on bacterial abundance or activity may be missed if not sampled at the adequate temporal resolution.

Bacterial consumption of DOM and inorganic nutrients depends on community composition because different bacterial taxa exhibit distinct metabolic capabilities and substrate preferences, and not all bacteria are equally active in carbon consumption, respiration or biomass production (Cottrell and Kirchman 2000; Alonso-Sáez and Gasol 2007; Ruiz-González *et al*. 2012; Sarmento and Gasol 2012). In turn, bacterial community composition and activity are strongly shaped by environmental gradients and biological factors such as viral infection or bacterivory, with some bacterial groups being more susceptible to these mortality factors than others (Boavida and Wetzel 1998; Bouvier and del Giorgio 2007; Mojica and Brussaard 2014; Teira *et al*. 2019). Responses of heterotrophic bacteria to SGD may hence be either direct (if responding to nutrient or carbon inputs or changes in conditions such as salinity) but also indirect if SGD has an influence on other planktonic organisms such as phytoplankton, viruses or bacterial predators. As an example, large but transient increases in the abundance and activity of opportunistic marine bacterial groups such as *Roseobacter* were observed upon experimental groundwater addition, which peaked in the first 24 h and decreased quickly afterward likely due to intense predation (Maister 2018).

Depending on the type of discharge, microbial responses to SGD may differ. Continuous diffusive SGD (Fig. 1A) may create stable gradients of groundwater influence, leading to the establishment of a succession in microbial communities like those observed along surface estuarine plumes (Bouvier and del Giorgio 2002; Troussellier *et al*. 2002). Point-sourced SGD inputs (Fig. 1B) or discharge peaks following precipitation events (Kim *et al*. 2003; Mejías *et al*. 2012), conversely, might trigger localized or ephemeral pulses of intense growth of taxa that may disproportionately contribute to carbon cycling, e.g. by channeling large amounts of carbon toward higher trophic levels. However, groundwater is hardly ever included as a factor shaping bacterial communities in coastal waters, highlighting the lack of interaction between microbial oceanographers and terrestrial ecologists or hydrogeologists.

### SGD-driven transport of microbial diversity into the coastal ocean

Given the high microbial diversity hidden in coastal aquifers and the magnitude of groundwater discharge at local and global scales, SGD may also influence coastal microbial communities through the transport of groundwater prokaryotes. In freshwater ecosystems, the terrestrial–aquatic connectivity of microbial assemblages has emerged as an important driver of microbial community composition and biogeography at the landscape scale (Langenheder and Lindström 2019). For example, lake and river bacterial assemblages are strongly impacted by the dispersal of microorganisms from the surrounding terrestrial landscape or upstream water bodies, some of which seem able to grow and dominate the aquatic environment (Crump, Amaral-Zettler and Kling 2012; Ruiz-González, Niño-García and del Giorgio 2015a; Niño-García, Ruiz-González and del Giorgio 2016). This SGD-driven microbial connectivity has mostly been addressed with regard to the transport of terrestrial pathogenic or fecal indicator bacteria (FIB, i.e. enterococci and *Escherichia coli*) through coastal aquifers or sediments, which can have health implications (Yau *et al*. 2014; Vollberg *et al*. 2019). Although some studies concluded that SGD may not be a main source of FIB (Knee *et al*. 2008), others reported a clear link between FIB and the concentration of SGD tracers such as radium isotopes (Paytan, Boehm and Shellenbarger 2004), as well as the presence of FIB and human enteric viruses sourced by groundwater even 10 km offshore (Futch, Griffin and Lipp 2010). Interestingly, different FIB can be transported from different sources (e.g. *E. coli* from sand washing and enterococci from groundwater; Russell *et al*. 2013) and different types of sediments can differentially retain fecal indicator bacteria or viruses (de Sieyes *et al*. 2016). Hence, processes operating at the land–ocean transition zone may modify not only the chemical but also the microbial composition of SGD.

SGD discharging to the ocean is presumably also loaded with the diverse microbial communities naturally inhabiting the subterranean estuary or coastal sediments (see the section 'Microbial abundance, diversity and environmental drivers along subterranean estuaries'). Several hydrogeological characteristics (e.g. groundwater velocity, type of aquifer and discharge, mechanisms driving the flow, degree of interaction between groundwater and seawater) may determine the identity of the aquifer microbes arriving to the ocean, as for example not all bacterial taxa are equally mobilized from intertidal sands by seawater (Boehm, Yamahara and Sassoubre 2014). To our knowledge, only two studies have suggested a potential role of SGD as conveyor of groundwater microorganisms from coastal aquifers into the marine environment: Lee *et al*. (2017) showed that both SGD flow and tidal fluctuation determined the periodic occurrence of freshwater-related bacteria in coastal bacterioplankton assemblages, and Menning *et al*. (2018) reported that changes in aquifer discharge controlled the microbial abundance and taxonomic richness in a spring-fed estuary.

It is still uncertain whether any of the groundwater or sediment prokaryotes transported via SGD can thrive in seawater, or whether marine taxa can survive within sandy sediments. However, there is experimental evidence that it is possible to retrieve living marine bacteria from sources such as freshwater, air or lake sediments (Comte *et al*. 2014; Langenheder *et al*. 2016) and that mixing of fresh- and saline water promotes the growth of initially rare taxa (Shen, Jürgens and Beier 2018; Rocca *et al*. 2020). Also, active freshwater bacterial taxa were detected at the marine extreme of an estuarine plume (Troussellier *et al*. 2002). These findings suggest that coastal terrestrial and freshwater landscape components could represent reservoirs of microbial taxonomic and functional diversity for marine communities, with yet unknown implications for marine community structure and functioning. The fact that most coastal groundwater microbial research has been focused on single aquifers, rarely including the adjacent seawater and disregarding the land–ocean microbial connectivity (Table S1, Supporting Information), has precluded establishing microbial singularities or commonalities across coastal aquifers or determining the magnitude of microbial fluxes between the aquifers and the sea.

## FUTURE DIRECTIONS AND RESEARCH AVENUES

**Figure 7.**
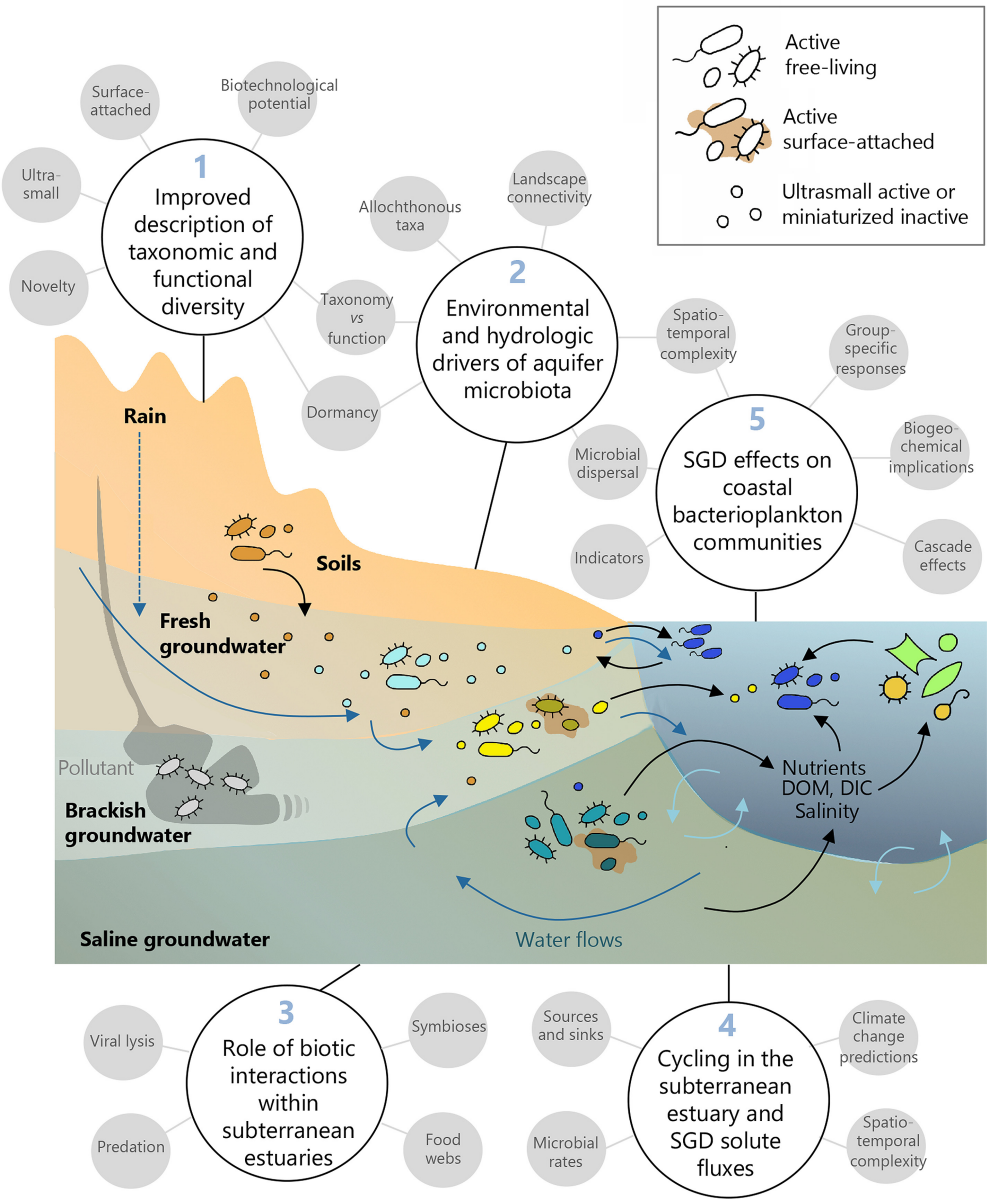
Future research directions to gain insight into the microbial dimension of SGD. Future studies should **(A)** improve the characterization of the taxonomic and functional diversity (including the ultrasmall, sediment-attached and previously unknown taxa) within subterranean estuaries; **(B)** elucidate the main environmental and hydrologic drivers of prokaryotic communities and their functions, considering the high spatiotemporal heterogeneity of these systems and features such as dormancy or dispersal of allochthonous taxa that may obscure links between community taxonomic composition (indicated by the color of prokaryotes), community functioning and external factors; **(C)** explore the role of biotic interactions (e.g. viruses, predators or symbiotic associations) and their implications for microbial community functioning; **(D)**incorporate knowledge on microbial processes and their drivers to constrain chemical SGD fluxes and their potential variations upon changes in conditions; and **(E)** gain insight into the responses of microbial communities to SGD, particularly bacterioplankton, focusing on the responses of specific microbial taxa, cascading effects and the dispersal of microorganisms from groundwater to the ocean. DOM, dissolved organic matter; DIC, dissolved inorganic carbon.

Deepening our knowledge of the microbial dimension at the land–ocean interface will be beneficial from multiple points of view, ranging from the discovery of novel species and metabolisms, to achieving more accurate predictions of SGD fluxes and their consequences in marine biogeochemical cycles. Based on the needs of the field identified in the previous sections, here we propose five future research avenues and strategies that may help researchers advance toward a better understanding of the microbial dimension of SGD (Fig. 7).

### Improving the description of microbial diversity and function in coastal aquifers

Given the high degree of taxonomic novelty found within single aquifers (Anantharaman *et al*. 2016), the study of the gene pools within both the active and the dormant fractions of communities from the undersampled coastal aquifers ensures the discovery of unimaginable living or surviving strategies, enriching current sequence catalogs (Fig. 7.1). PCR-free sequencing techniques like metagenomics, metatranscriptomics or single cell genomics (Appendix 2) have uncovered an enormous variety of novel taxa and metabolisms in inland aquifers that are not captured by current primer-based approaches (Jewell *et al*. 2016; Lau *et al*. 2016; Probst *et al*. 2017, 2018; Bell *et al*. 2018), so their application to coastal aquifers will offer key insight into the functional potential hidden in these systems. The ongoing accumulation of genomic information from inland aquifers will facilitate the application of these techniques to coastal aquifer microbiota by providing reference genomes and sequences. Moreover, metagenomic information may also help in the design of new PCR taxonomic or functional primers (e.g. Cornejo-Castillo 2017), which could be optimized for groundwater prokaryotic diversity. Applied to the ultrasmall fraction of groundwater communities (<0.2 µm), these approaches will shed light on the unknown role of the minute *Ca*. Patescibacteria or the DPANN Archaea in subterranean estuaries.

Techniques that allow linking metabolic processes and activity rates to specific microbial groups offer ways to improve our understanding of the role of different prokaryotic groups in the cycling of substrates in coastal aquifers. For example, SIP of DNA can identify microorganisms involved in the use of different compounds (labeled with stable isotopes) through the selective recovery of heavy isotope-enriched DNA: The microbial taxa involved in autotrophic fixation of CO_2_ (Lazar *et al*. 2017) or in the degradation of plant-derived polysaccharides (Taubert *et al*. 2019) were identified in inland aquifers applying SIP. Also, SIP coupled to Raman microspectroscopy (Raman-SIP) unveiled the groundwater bacterial populations activated by different plant-derived compounds using heavy water (D_2_O) as a tracer (Taubert *et al*. 2018) and demonstrated complete denitrification by a groundwater microbial community adding ^15^NO_3_^−^ (Kumar *et al*. 2018).

The relative contribution of different microbial taxa to substrate uptake can also be elucidated at the single-cell level through techniques such as microautoradiography (Brock 1967) or BONCAT (bioorthogonal non-canonical amino acid tagging) (Hatzenpichler *et al*. 2014), which combined fluorescence *in situ* hybridization techniques (FISH or CARD-FISH; Appendix 2) allow the visual identification of different active prokaryotic taxa. Microautoradiography has widely been used to quantify the uptake of different radiolabeled compounds by bacterial groups in freshwater and marine systems (Pérez and Sommaruga 2006; Ruiz-González *et al*. 2012), but only rarely in groundwater (Wilhartitz *et al*. 2009; Kellermann *et al*. 2012). More recently developed, BONCAT is based on the fluorescent labeling of the newly synthesized proteins. Combined with CARD-FISH or flow cytometry (Sebastián and Gasol 2019), it allows visually identifying active cells within specific taxonomic groups, making it a promising tool for understanding the metabolic status of microbial inhabitants in coastal aquifers (Maister, 2018).

**Figure 8.**
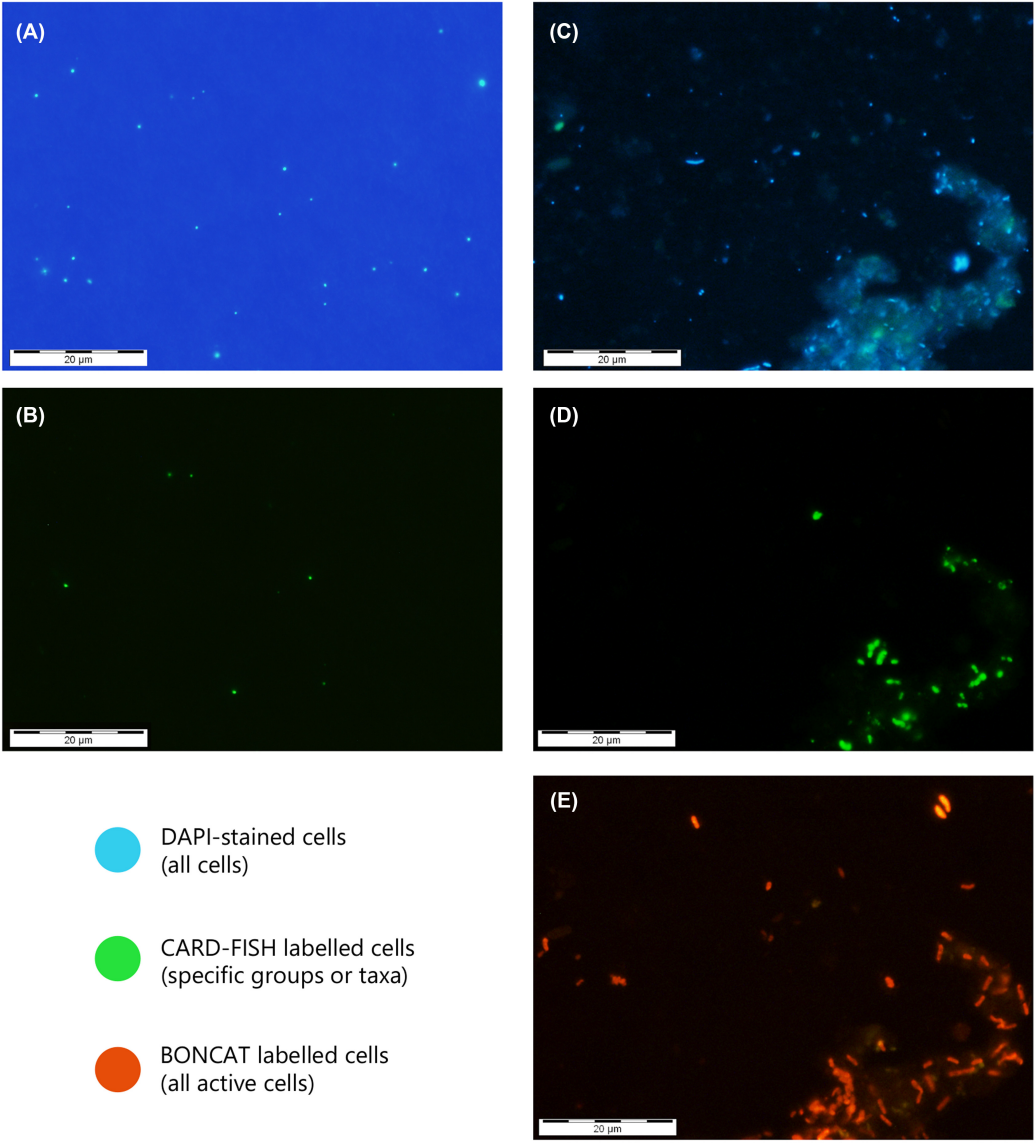
Visualization is essential to gain insight into the ecology of different groundwater microbial groups. Examples of microscopy pictures showing the presence of ultrasmall *Ca*. Parcubacteria **(A, B)** or large Gammaproteobacteria **(C–E)** in two Mediterranean coastal aquifers in Spain [a limestone-karstified coastal aquifer (Garcia-Solsona *et al*. 2010) and an alluvial aquifer (Folch *et al*. 2020), respectively]. Micrographic images show: (A, C) all microbial cells stained with 4′,6-diamidino-2-phenylindole (DAPI); specific bacterial groups hybridized with the CARD-FISH probes OD1-289 (Gong *et al*. 2014) for Parcubacteria clades (B), and Gamma42a (Manz *et al*. 1992) for Gammaproteobacteria (D). (E) Translationally active cells (detected with BONCAT, see the section 'Improving the description of microbial diversity and function in coastal aquifers'). Scale bars are 20 µm. Note the large difference in size between both groups, with obvious implications for their contributions to total biomass and activity, information that cannot be derived from sequencing approaches. Gammaproteobacteria (D) seem to be mostly concentrated on particles, as well as the majority of active cells (E).

The visualization of microorganisms and their activity is actually becoming increasingly needed in an era dominated by sequencing technologies, as it provides essential information on microbial processes or features that cannot be derived from sequencing data (Sebastián and Gasol 2019). For example, size-fractionated filtration for DNA sequencing does not allow unequivocally determining whether a given bacterial taxon is actually small, as filters may clog and retain small cells. Indeed, we have found cells identified as ultrasmall *Ca*. Parcubacteria (see the section 'Ultrasmall prokaryotic groups are abundant in groundwater ecosystems') onto the 0.2-µm filter in a coastal alluvial aquifer (Fig. 8A and B), which, to our knowledge, represents the first observation of ultrasmall cells in coastal saline groundwater. The comparison with the large Gammaproteobacteria detected in another subterranean estuary (Fig. 8C and D) highlights the relevance of visualization for understanding the contribution of the different groundwater microbial groups to total biomass, for learning about their lifestyles (free-living, attached), or for deciphering their contribution to community activity (Fig. 8E; Sebastián and Gasol 2019).

Finally, the sampling of prokaryotes attached to surfaces should also be implemented in parallel to water sampling to achieve the full picture of the groundwater microbiota (Smith *et al*. 2018). Whereas characterizing the taxonomic composition of microbial communities in unconsolidated sediments is relatively easy if the sediment can be accessed, estimating the microbial biomass and activity associated with these surfaces remains challenging (Smith *et al*. 2018). Recent protocols for the direct quantification of prokaryotic abundance in groundwater sandy sediments (Bayer *et al*. 2016) and the combination of traditional activity assays with new sequencing techniques performed at scales relevant for microbial processes (Smith *et al*. 2018) open the door for the simultaneous assessment of the two microbial compartments.

### Expanding knowledge of chemical and hydrologic drivers of microbial communities in subterranean estuaries

Most current information on groundwater microbial diversity (in particular that from coastal aquifers) comes from snapshot observations in specific locations (Table S1, Supporting Information), so we lack a comprehensive view of the large-scale spatial patterns and the temporal dynamics of the microbial communities in subterranean estuaries. Comparative studies covering large environmental and spatial gradients are needed to gain insight into the most important drivers of groundwater microbial communities and their functions (Fillinger, Hug and Griebler 2019a) but also studies exploring the highly dynamic spatiotemporal heterogeneity within single coastal aquifers (McAllister *et al*. 2015; Menning *et al*. 2018; Jiang *et al*. 2020; Fig. 7.2). This requires overcoming the technical limitations described previously, such as optimizing primer diversity coverage and reaching a consensus on which primers to use to be able to compare among groundwater studies (see the sections 'Novel groundwater microbial taxa can be missed by current sequencing technologies' and 'Improving the description of microbial diversity and function in coastal aquifers'). The lower cost and higher detection of rare prokaryotic taxa by primer-based approaches than by metagenomics/metatranscriptomics make them useful tools to gain insight into the mechanisms of assembly and drivers of groundwater communities across large numbers of samples. In addition, metagenomic and metatranscriptomic analyses require much more initial biomass than primer-based sequencing, which may be logistically challenging due to the low microbial abundances typically observed in groundwater (e.g. 400–800 L was filtered for reconstructing genomes of ultrasmall cells by He *et al*. 2021).

Being able to distinguish between the truly active bacteria from those dormant, dead or allochthonous will also improve our understanding of the role of different drivers on coastal groundwater microbial communities. Experimental manipulations to promote or detect increases in the abundances of rare or dormant taxa (Rajala and Bomberg 2017; Hofmann and Griebler 2018; Fig. 5), sequencing the RNA instead of the DNA to target the potentially active community (Aanderud *et al*. 2016; Wisnoski *et al*. 2020), quantification of relic DNA (Carini *et al*. 2016; Lennon *et al*. 2018), or approaches that allow linking taxonomy with actual microbial activity (see the section 'Improving the description of microbial diversity and function in coastal aquifers') offer ways to learn about the metabolic status and role of different microbial taxa at the land–ocean transition zone.

Flow cytometry may also be used to readily characterize the physiological status of cells along the land–ocean transition zones, such as through the nucleic acid double-staining protocol (Grégori *et al*. 2001) that allows distinguishing dead or damaged cells from healthy cells in planktonic samples, or the detection of actively respiring cells stained with 5-cyano-2,3-ditolyl tetrazolium chloride (Sieracki, Cucci and Nicinski 1999). Differentiating high versus low nucleic acid content cells (HNA vs LNA) may help estimate variations in ultrasmall planktonic cells along environmental gradients (Proctor *et al*. 2018).

Considering the bidirectional dispersal of groundwater and marine species is also critical, as it may impact aquifer functioning inoculating viable taxa in both directions, or mask relevant patterns by delivering significant amounts of inactive allochthonous taxa. Microbial source tracking methods, which allow assigning the most plausible habitat to a given taxon in order to infer potential dispersal sources (Comte *et al*. 2017; Ortiz-Álvarez *et al*. 2019) could allow defining connectivity pathways within aquifers and tracking the abundances of potentially inactive allochthonous taxa (e.g. Fillinger *et al*. 2021). The quantification of endospores as done in deep marine sediments (Wörmer *et al*. 2019) or of genes involved in dormancy (Lennon and Jones 2011) may further help assess the extent of dormancy and the factors governing activity in these dynamic environmental transitions. Importantly, for any of these microbiological investigations to yield accurate insight, they must be accompanied by detailed hydrogeological assessments of the relevant characteristics of the system, including the physical forces driving the flow, the dynamic movement of the fresh–saline interface, or the groundwater residence time in the subterranean estuary.

### Elucidating the role of biotic interactions as drivers of microbial communities in coastal aquifers

Besides environmental or hydrologic factors, biotic interactions are emerging as key drivers of food webs and biogeochemical cycles in ecosystems such as the ocean (Lima-Méndez *et al*. 2015; Wilkins *et al*. 2019), but little is known about the interactions between indigenous microorganisms in the groundwater environment (Fig. 7.3). Some studies in inland aquifers have suggested that planktonic prokaryotes are more strongly controlled by viral lysis than by bacterivores (Wilhartitz *et al*. 2013), and that viruses can influence carbon biogeochemistry and prokaryotic community structure (Pan *et al*. 2014). Predation by flagellates was shown to be intense and size selective in a contaminated aquifer (Kinner *et al*. 1998) and predatory bacteria (i.e. bacteria that feed on other bacteria) were isolated from a coastal alluvial aquifer (Banning, Casciotti and Kujawinski 2010), yet the relevance of this mechanism in shaping prokaryotic communities is largely unknown. Interestingly, experimental exposure to protozoan grazers caused changes in sediment-associated bacterial communities and increased the retention of ^13^C-labeled organic carbon in groundwater sediments (Longnecker and Kujawinski 2013). This suggests that these biotic interactions may impact not only the microbial structure of communities but also biogeochemical processes in both the planktonic and the sediment-attached compartments.

Recently, some multi-approach studies have started to shed light into the complexity of trophic interactions within subsurface ecosystems. Combining isotopic composition of food resources and consumers with information on food web structure, predator species diversity and organic matter, Hutchins *et al*. (2016) unveiled that chemolithoautotrophic organic matter production supported complex macroinvertebrate food webs and their species diversity in a groundwater system. Similarly, a combination of geochemical, genomic and biomarker approaches revealed that carbon from methanotrophic bacteria supported a large fraction of the diet of cave-adapted shrimps in a tropical subterranean estuary (Brankovits *et al*. 2017), highlighting the relevance of chemolithoautotrophic metabolisms for sustaining life in these coastal systems.

Microscopy may also help elucidate biological interactions within coastal groundwater microbial assemblages. Cryotransmission electron microscopy (cryo-TEM) was essential to determine that ultrasmall cells in an inland aquifer were metabolically active rather than starved and dormant, as they appeared infected by viruses or physically interacting with other bacterial cells, further indicating a potential symbiotic lifestyle (Luef *et al*. 2015; He *et al*. 2021). Also, using genomic information to design FISH probes, Schwank *et al*. (2019) uncovered a host–symbiont association between two uncultivated archaeal phyla in a deep aquifer. Due to the small genome of many of the novel phyla discovered in groundwater, it has been hypothesized that many groundwater groups, including members within *Ca*. Patescibacteria, may have a symbiotic lifestyle (Nelson and Stegen 2015; Youssef *et al*. 2015, but see Beam *et al*. 2020). This, coupled to the genomic evidence that groundwater microbial community members are intimately linked through the exchange of chemical substrates (Anantharaman *et al*. 2016; Lau *et al*. 2016), and that most groundwater biomass is in the form of biofilms where cells are in close contact to one another (Smith *et al*. 2018), suggests that symbiotic relationships (either through obligate or facultative syntrophy, mutualism or commensalism) might be essential in sustaining the microbiome and biogeochemical cycling in groundwater ecosystems. The study of biotic interactions within subterranean estuaries is, to our knowledge, a largely unexplored but promising field of research.

### Incorporating microbial processes to constrain and better estimate SGD fluxes

Integrating the microbial perspective into SGD studies should improve our capacity to constrain the magnitude of SGD-driven solute fluxes and their ecological implications (Fig. 7.4). Accurate estimates of solute fluxes supplied by SGD (at local, regional and global scales) require understanding the chemical transformations occurring to groundwater before it reaches the sea, including the chemical cycling controlled by microbial communities. Knowledge of microbial ecology in the subterranean estuary thus needs to be coupled with estimates of biogeochemical and hydrogeological processes to unravel the role of subterranean estuaries in controlling solute fluxes to the ocean; this is actually one of the major knowledge gaps in the SGD field highlighted by recent reviews (Robinson *et al*. 2018; Taniguchi *et al*. 2019).

Most knowledge about element cycling in subterranean estuaries has been achieved by piecing together physicochemical snapshots obtained from profiles along cross-shore transects, and by comparing nutrient concentrations in the fresh and marine endmembers (e.g. Gonneea *et al*. 2014; Reckhardt *et al*. 2017). While these evaluations provide key information on the behavior of chemicals in the subterranean estuary, they do not always allow identifying the controlling mechanisms or predicting the potential consequences due to changes in the environment (because different processes may yield similar chemical snapshots). For example, coastal aquifers are especially sensitive to salinization (i.e. seawater intrusion) due to both sea level rise and excessive withdrawal of water for human purposes (Ferguson and Gleeson 2012; Werner *et al*. 2013; Retter, Karwautz and Griebler 2021) and hence the salinity tolerances of different microbial taxa will control how a given process respond to the expected salinity changes. Actually, seawater intrusion has been shown to impact the distribution and activity of microbial communities in coastal peat deposits, resulting in increases in the release of greenhouse gases (CH_4_, CO_2_) driven by SGD (Kreuzburg *et al*. 2020). Salinity increases might also enhance the delivery of NH_4_^+^ from groundwater to the coast due to increased N mineralization and decreased nitrification (Santoro 2010). Investigations in anchialine systems (i.e. coastal sinkholes with a subterranean connection with the ocean; see Fig. 1B) might shed light on some of the processes and the drivers operating in coastal underground ecosystems; their higher accessibility may allow a coverage of the spatial heterogeneity in environmental gradients and/or microbial dynamics that is not feasible in most subterranean estuaries (e.g. Seymour *et al*. 2007; González *et al*. 2011) or the sampling and functional exploration of surface-attached microbial communities (Haas *et al*. 2018).

In turn, prospection of subsurface microbial genes and metabolisms might help counteract our impact on groundwater by means of microbially mediated management practices, like for example the degradation of pollutants (Posman, DeRito and Madsen 2017) or those enhancing nitrogen removal from aquifers (Ludington *et al*. 2017). Denitrification has long been considered as the main process removing nitrogen from coastal aquifers, but other less studied metabolisms performed by largely unknown microbial players, e.g. DNRA and anammox, seem to be important pathways of nitrogen loss in aquifers globally (Wang *et al*. 2020). In any case, it will not be possible to accurately interpret microbial data without a good knowledge of the system (geological setting, geochemistry, hydrology). Attempts to join different approaches such as nutrient mass-balances, stable isotopic signatures of water and sediments, information on DOM composition, redox conditions, water residence time and microbial communities have provided useful insight into the different sources of chemical elements, the complexity of subsurface biogeochemical transformations and resulting SGD-driven solute fluxes (Beck *et al*. 2017; Montiel *et al*. 2019). Therefore, only through multidisciplinary efforts we may achieve a holistic view of the biotic interactions and biogeochemical processes within coastal aquifers.

### Deepening our understanding of SGD effects on marine food webs and biogeochemical cycles

Studies exploring the effects of SGD on the microbial components at the base of food webs (including bacterioplankton) will provide useful insight into the ecological and biogeochemical consequences of SGD in the marine environment, improving our understanding of the microbial regulation of nutrient, carbon and metal cycles in the coastal ocean (Fig. 7.5). As the element fluxes differ largely depending on the characteristics of the aquifer (e.g. natural and anthropogenic inputs into groundwater, geologic characteristics and type of discharge, water flow velocity; Tovar-Sánchez *et al*. 2014), an accurate understanding of the microbial responses to SGD necessarily requires detailed evaluations of the magnitude of SGD-driven chemical fluxes.

Coupling microbial dynamics with estimations of SGD-driven solute fluxes derived from chemical tracers (e.g. radium isotopes, radon, methane, silica; Dulaiova *et al*. 2010; Rodellas *et al*. 2017; Oehler *et al*. 2019) or the application of mixing models along salinity gradients (as in Stegen *et al*. 2016) may be useful to identify and quantify localized microbial responses to SGD. Experimentation could allow differentiating between direct SGD effects on microbial communities (through the acquisition of SGD solutes or changes in the environment), or indirect cascade effects such as bacterial responses to SGD-driven changes in phytoplankton production of DOM or grazer or viral activity. Isotopic fractionation may provide valuable insight on the transfer of nutrients or carbon from groundwater into different marine microbial compartments (e.g. primary producers; Andrisoa *et al*. 2019), allowing to estimate the fate of the delivered elements in coastal ecosystems. The degree of allochthony in aquatic bacteria (i.e. the contribution of terrestrial dissolved organic carbon to aquatic bacterial biomass) has been extensively studied in freshwater systems (e.g. Guillemette, McCallister and del Giorgio 2016), yet to our knowledge this has never been explored in coastal bacterial communities influenced by SGD. Finally, microbial transformations controlling dissolved organic matter (DOM) quality within coastal aquifers (Chaillou, Lemay-Borduas and Couturier 2016; Couturier, Nozais and Chaillou 2016; Jiang *et al*. 2020) may in turn determine the taxonomic or functional responses of planktonic marine bacterioplankton influenced by SGD, given that DOM quality is a main driver of aquatic bacterioplankton structure and functioning (Landa *et al*. 2014; Ruiz-González *et al*. 2015b). Applying the above-mentioned techniques to link taxonomy with function (see the section 'Improving the description of microbial diversity and function in coastal aquifers') along natural groundwater gradients or upon experimental groundwater additions will help identify specific responses of different marine bacterial groups to SGD.

On the other hand, the influence of the dispersal of groundwater microbial communities on coastal ecosystems could be resolved by means of experimental assessments of the capacity of groundwater microorganisms to thrive in marine conditions (e.g. mixing water types; Maister 2018) or through transplant experiments (Shen, Jürgens and Beier 2018), or by tracking the presence of groundwater taxa in marine sites. Microbial source tracking methods (see the section 'Expanding knowledge of chemical and hydrologic drivers of microbial communities in subterranean estuaries') have been applied to identify the origin of fecal bacteria in the surf zone (Russell *et al*. 2013) and could be used to detect groundwater taxa in coastal areas (or vice versa) if simultaneously characterizing coastal aquifers and marine sites (Unno *et al*. 2015; Adyasari *et al*. 2019). Specifically, the SGD-driven transport of taxa able to thrive in coastal seawater might represent a diversity pathway not previously considered, with potential implications for marine community structure and functioning. Finally, whether viruses, bacteria or protists delivered with coastal groundwater can directly interact with marine bacterioplankton communities remains completely unknown.

Groundwater taxa arriving to the sea may be used as microbial 'indicators' or tracers of groundwater inputs to the coastal zone. Recent studies have used microbial information to identify tidal or temporal variations of SGD (Lee *et al*. 2017), to distinguish groundwater recharge sources to shallow wells (Higgings *et al*. 2020), to uncover sporadic episodes of intense groundwater influence (Kim *et al*. 2020) or to detect contamination episodes, given that specific taxa can be used as biosensors even after a given pollutant has been fully degraded (Smith *et al*. 2015). Similarly, the presence of marine microbial taxa in coastal aquifers can indicate active land–ocean connectivity, and bacterial taxa within Oceanospirillales and Alteromonadaceae (Chen *et al*. 2019a) or within Rhodobacteraceae and Flavobacteriaceae (Unno *et al*. 2015) have been proposed as potential indicators of seawater intrusions in coastal aquifers. The identification of pathogens or fecal bacteria may further allow the detection of wastewater contamination in coastal aquifers or in the adjacent seawater (Boehm, Shellenbarger and Paytan 2004; Yau *et al*. 2014; Ahmed *et al*. 2019). As many of these 'indicators' may not survive in marine systems, their detection will refine our capacity to distinguish between allochthonous and truly marine taxa.

## CONCLUDING REMARKS

The submarine discharge of groundwater to the coastal ocean constitutes a terrestrial and marine linkage of high relevance for the functioning of marine ecosystems, which is modulated to a large extent by the microbial processes occurring in the coastal aquifer. Despite the growing interest on groundwater microbiology in recent years, coastal aquifers have received little attention from microbial studies in comparison to surface waters. In turn, SGD research has been historically dominated by studies performed from either a 'chemical oceanography' or a 'hydrological' point of view, lacking a biological understanding of the underlying processes.

Here, we have identified some of the current challenges and future directions that should be tackled for a deeper understanding of the microbial dimension underlying SGD processes. These include improving the characterization of groundwater microbiota by overcoming current technical limitations and considering the specific characteristics of coastal aquifers (i.e. dynamic mixing of water, chemicals and communities). In turn, the evaluation of solute fluxes (e.g. nutrients, metals, carbon, contaminants) supplied by SGD needs to rely on an accurate understanding of the microbially mediated chemical cycling in the coastal aquifer, requiring an improved knowledge on their drivers and metabolic capacities and responses. Finally, coastal marine microbial ecology needs to incorporate the connectivity with the continent, and in particular this largely overlooked groundwater linkage; good knowledge of the hydrogeology and the chemical and microbial fluxes from the coast to the ocean will expand our understanding of the structuring and function of coastal planktonic communities, as well as the ecological consequences of SGD. Only by moving beyond the presumed limits of ecosystems and research fields (Xenopoulos *et al*. 2017; Kayler *et al*. 2019), we may obtain a comprehensive understanding of the interlinked physical and biogeochemical processes occurring throughout the terrestrial–marine continuum.

## ACKNOWLEDGMENTS

Particular thanks to Marta Sebastián and Andrea G. Bravo for their useful and constructive comments on the manuscript, and to Josep M. Gasol, Olena Maister, Marc Diego-Feliu and Aaron Alorda-Kleinglass for their help during some of the first exploratory field surveys. We acknowledge support of the publication fee by the CSIC Open Access Publication Support Initiative through its Unit of Information Resources for Research (URICI).

## FUNDING

This work was funded by the Spanish Ministry of Science, Innovation and Universities (MICINN) through the GRAMMI project (RTI2018-099740-J-I00) and partly by the projects MEDISTRAES II (CGL2016-77122-C2) and OPAL (PID2019-110311RB-C21). Additional financial support was provided by grants SPIP2020-02595 (Ministry for the Ecological Transition and the Demographic Challenge (MITECO)) and 2017SGR/156 (Generalitat de Catalunya), with funding from the Spanish government through the ‘Severo Ochoa Centre of Excellence’ accreditation (CEX2019-000928-S).VR acknowledges financial support from the Beatriu de Pinós postdoctoral programme of the Catalan Government (2017-BP-00334). This study contributes to the work carried out by the MERS research group 2017 SGR 1588.

## Supplementary Material

fuab010_Supplemental_Files
