# Supplementary material for: The microbial dimension of submarine groundwater discharge: current challenges and future directions
Source: FEMS Microbiol Rev. 2021 Feb 4;45(5):fuab010. doi: 10.1093/femsre/fuab010 (PMC8498565; doi:10.1093/femsre/fuab010)
Supplement: fuab010_Supplemental_Files [file fuab010_supplemental_files.zip › Table_S2.docx]

**Table S2.** Sequences of the 13 different primer pairs used by the 25 studies included in Table S1 that described the prokaryotic diversity within coastal aquifers. ‘Target group’ indicates whether the primers retrieve diversity within the domains Bacteria, Archaea, or Eukarya based on the results of the Test Prime analysis (see Figure 3). Note that Chen *et al.* 2019b used two different sets of primers.

| **Study** | **Primer pair** | **Target group** | **Primer forward (5’-3’)** | **Primer reverse (5'-3')** |
| --- | --- | --- | --- | --- |
| Santoro et al. 2008 | 21F-958R | Archaea | TTCCGGTTGATCCYGCCGGA | YCCGGCGTTGAMTCCAATT |
| Rogers and Calciotti 2010 | 21Fa-1492R | Archaea | TTCCGGTTGATCCYGCCGGA | GGTTACCTTGTTACGACTT |
| Chen et al. 2019b | 524F10extF-Arch958RmodR | Archaea | TGYCAGCCGCCGCGGTAA | YCCGGCGTTGAVTCCAATT |
| Banning et al. 2010 | 27F-1492R | Bacteria | AGAGTTTGATCCTGGCTCAG | GGTTACCTTGTTACGACTT |
| González et al. 2011 | 27F-1492R | Bacteria | AGAGTTTGATCCTGGCTCAG | GGTTACCTTGTTACGACTT |
| Haas et al. 2018 | 27F-1492R | Bacteria | AGAGTTTGATCCTGGCTCAG | GGTTACCTTGTTACGACTT |
| Sola et al. 2020 | 341F-785R | Bacteria | CCTACGGGNGGCWGCAG | GACTACHVGGGTATCTAATCC |
| Chen et al. 2019a | 341F-806R | Archaea/Bacteria | CCTAYGGGRBGCASCAG | GGACTACNNGGGTATCTAAT |
| Chen et al 2020a | 341F-806R | Archaea/Bacteria | CCTAYGGGRBGCASCAG | GGACTACNNGGGTATCTAAT |
| Davis and Garey 2018 | pro341F-pro805R | Archaea/Bacteria | CCTACGGGNBGCASCAG | GACTACNVGGGTATCTAATCC |
| Beck et al. 2017 | 357F-907R | Bacteria | CCTACGGGAGGCAGCAG | CCGTCAATTCCTTTRAGTTT |
| Héry et al. 2014 | 43F-806R | Bacteria | TACGGRAGGCAGCAG | GGACTACCAGGGTATCTAAT |
| Unno et al. 2015 | 515F-806R | Archaea/Bacteria | GTGCCAGCMGCCGCGGTAA | GGACTACHVGGGTWTCTAAT |
| Brankovits et al. 2017 | 515F-806R | Archaea/Bacteria | GTGCCAGCMGCCGCGGTAA | GGACTACHVGGGTWTCTAAT |
| Hong et al. 2019 | 515F-806R | Archaea/Bacteria | GTGCCAGCMGCCGCGGTAA | GGACTACHVGGGTWTCTAAT |
| Missimer et al. 2014 | 515F-806R | Archaea/Bacteria | GTGCCAGCMGCCGCGGTAA | GGACTACHVGGGTWTCTAAT |
| Sang et al. 2019 | 515F-806R | Archaea/Bacteria | GTGCCAGCMGCCGCGGTAA | GGACTACHVGGGTWTCTAAT |
| Sang et al. 2018 | 515F-806R | Archaea/Bacteria | GTGCCAGCMGCCGCGGTAA | GGACTACHVGGGTWTCTAAT |
| Zhang et al. 2021 | 515F-806R | Archaea/Bacteria | GTGCCAGCMGCCGCGGTAA | GGACTACHVGGGTWTCTAAT |
| Ye et al. 2016 | 515F-907R | Bacteria/Eukaryota | GTGCCAGCMGCCGCGG | CCGTCAATTCMTTTRAGTTT |
| Chen et al. 2019b | 515F-907R | Bacteria/Eukaryota | GTGCCAGCMGCCGCGG | CCGTCAATTCMTTTRAGTTT |
| Chen et al. 2020b | 515F-907R | Bacteria/Eukaryota | GTGCCAGCMGCCGCGG | CCGTCAATTCMTTTRAGTTT |
| Adyasari et al. 2019 | 515YF-926R | Archaea/Bacteria/Eukaryota | GTGYCAGCMGCCGCGGTAA | CCGYCAATTYMTTTRAGTTT |
| Montiel et al. 2019 | 515YF-926R | Archaea/Bacteria/Eukaryota | GTGYCAGCMGCCGCGGTAA | CCGYCAATTYMTTTRAGTTT |
| Adyasari et al. 2020 | 515YF-926R | Archaea/Bacteria/Eukaryota | GTGYCAGCMGCCGCGGTAA | CCGYCAATTYMTTTRAGTTT |
| Teng et al. 2014 | PRBA338F-PRUN518R | Bacteria | ACTCCTACGGGAGGCAGCAG | ATTACCGCGGCTGCTGG |
